# Coptisine Alleviates Pristane-Induced Lupus-Like Disease and Associated Kidney and Cardiovascular Complications in Mice

**DOI:** 10.3389/fphar.2020.00929

**Published:** 2020-06-19

**Authors:** Yu Yan, Zhihui Zhang, Yucai Chen, Biyu Hou, Kang Liu, Hailin Qin, Lianhua Fang, Guanhua Du

**Affiliations:** ^1^State Key Laboratory of Bioactive Substances and Functions of Natural Medicines, Institute of Materia Medica, Chinese Academy of Medical Sciences and Peking Union Medical College, Beijing, China; ^2^Department of Pharmacy, China-Japan Friendship Hospital, Beijing, China; ^3^School of Traditional Chinese Medicine, Beijing University of Chinese Medicine, Beijing, China; ^4^Department of Pharmacy, Electric Power Teaching Hospital, Capital Medical University, Beijing, China

**Keywords:** coptisine, mice, systemic lupus erythaematosus, Rho-associated coiled-coil-containing protein kinase, cardiovascular complication, renal complication

## Abstract

Systemic lupus erythaematosus (SLE) is a chronic multi-system autoimmune disease with a high prevalence of kidney and cardiovascular complications. Considering that Rho-associated coiled-coil-containing protein kinases (ROCKs) play important roles in SLE, inflammation, and cardiovascular disease, we hypothesized that coptisine, which has been found to inhibit ROCKs, may have an effect on SLE. The effect of coptisine was assessed in female BALB/c mice intraperitoneally injected with 0.5 mL of pristane. Serum autoantibodies were tested every month, blood pressure was measured every 2 months, and serum inflammatory markers, spleen pathologic characteristics, renal injury and vascular function were observed at 6 months. The results showed that coptisine decreased the levels of serum autoantibodies and serum inflammatory markers in the SLE mice, improved the pathologic characteristics of the spleen, and simultaneously improved renal injury, decreased inflammatory responses in the kidneys, reduced blood pressure, and improved vascular endothelial function. Western blot assays revealed that inhibiting the activation of the NF-κB and Rho/ROCK signalling pathways and downstream signalling molecules might be the potential mechanisms of the effects of coptisine. Our findings suggest that therapy with coptisine may be a strategy for preventing SLE and ameliorating associated kidney and cardiovascular complications.

## Introduction

Systemic lupus erythaematosus (SLE) is a systematic autoimmune disease characterized by immune complex deposition, autoantibody production, and multi-organ damage. SLE is associated with significant morbidity and mortality driven by infectious, renal, and cardiovascular complications (Ocampo-Piraquive et al., 2018; Mageau et al., 2019). The general consensus is that kidney involvement in the form of immune complex glomerulonephritis, which can ultimately lead to renal failure and even death, occurs in more than 50% of patients with SLE (Venegas-Pont et al., 2009; Mu et al., 2017). In addition to prominent renal disease, another primary cause of mortality in SLE is cardiovascular disease. Studies have reported that women with SLE are at a 50-fold greater risk for developing cardiovascular disease independent of traditional Framingham Heart Study risk factors (Munguia-Realpozo et al., 2019). To date, existing common therapies for SLE, namely, corticosteroids and anti-malarial and various immunosuppressive agents, despite being effective in suppressing disease progression, rarely offer long-term remission; moreover, medication-related toxicity contributes to morbidity and mortality, and existing drugs have little effect on kidney and cardiovascular complications.

Rho-associated coiled-coil-containing protein kinases (ROCKs) are the downstream targets of the small GTP-binding protein RhoA (Wettschureck and Offermanns, 2002). RhoA/ROCK signalling regulates numerous cell functions, including contraction, cell proliferation, migration, adhesion, apoptosis, cell cycle progression, and gene expression (Dong et al., 2010). Emerging evidence suggests that aberrant ROCK activation leads to abnormal levels of Ezrin/Radixin/Moesin (ERM) phosphorylation, which is one of the cytoskeletal abnormalities detected in T cells from SLE patients (Li et al., 2007). Murine studies have demonstrated that ROCK2 is activated in Th17 cells and phosphorylates interferon regulatory factor 4 (IRF-4), thus regulating the production of interleukin (IL)-17 and IL-21, two cytokines known to contribute to the pathogenesis of lupus (Biswas et al., 2010). The administration of the nonselective ROCK inhibitor fasudil has been shown to ameliorate the deregulated production of IL-17 and IL-21, as well as the inflammatory and autoantibody responses observed in MRL/lpr and NZB/W F1 mice (Stirzaker et al., 2012). Further research has shown that ROCK activity is elevated in peripheral blood mononuclear cells (PBMCs) from patients with SLE and RA and that the production of IL-17 and IL-21 by SLE T cells or Th17 cells can be inhibited by targeting the RhoA/ROCK pathway *via* both non-selective and selective approaches (Rozo et al., 2017). Therefore, ROCK inhibitors may be potential effective small molecules for the treatment of SLE.

In our previous studies, coptisine was found to inhibit ROCKs (Gong et al., 2012b; Guo et al., 2013). Coptisine, a naturally occurring isoquinoline alkaloid, is a bioactive constituent of the dried rhizome of *Coptis chinensis* Franch. In previous studies, several biological activities of coptisine were reported, including anti-inflammatory, anti-hypercholesterolemia, vasodilation, and cardioprotective properties (Gong et al., 2012a; Gong et al., 2012b; Lee et al., 2012; Guo et al., 2013; He et al., 2015; Zou et al., 2015). However, to date, the effects of coptisine on SLE and pristane-induced lupus have not been explored. Therefore, in this study, we evaluated whether coptisine could prevent the development of pristane-induced lupus in a mouse model and whether it can protect the kidneys and lower cardiovascular risk.

## Methods

### Reagents

Coptisine (**Figure S1**) was extracted by the Department of Medicinal Chemistry of our institute, and its structure was confirmed by the analysis of physical and chemical properties and spectral evidence. Pristane, norepinephrine (NE), phenylephrine (PE), acetylcholine (ACh), sodium nitroprusside (SNP), dihydroethidium (DHE), calf thymus double-stranded DNA (dsDNA), total calf thymus histone, goat anti-mouse IgG, and bull serum albumin (BSA) were purchased from Sigma-Aldrich Co. (St. Louis, MO, USA). Smith (Sm) antigen was purchased from RayBiotech, Inc. (Norcross, GA, USA). TMB substrate and RIPA Buffer (10×) were purchased from Cell Signaling Technology Inc. (Beverly, USA). BCA protein assay kit was purchased from Applygen Technologies Inc. (Beijing, China). Enhanced chemiluminescence reagent kit was purchased from CWBIO (Beijing, China). All other reagents were of analytical purity. Information regarding antibodies used in this study are listed in **Table S1**.

### Animals

BALB/c mice [female, 8 weeks, 18 ± 2 g, certificate no. SCXK (Beijing) 2012-001] were purchased from Vital River Laboratories (Beijing, China). The animals were maintained in a barrier system with an alternating 12 h light/dark cycle, a relative humidity of 50 ± 5% and a constant temperature of 24°C. All experimental protocols involving the care and use of the animals were reviewed and approved by the Laboratories Institutional Animal Care and Use Committee of the Chinese Academy of Medical Sciences and Peking Union Medical College.

### Experimental Protocols

BALB/c mice were intraperitoneally injected with 0.5 mL of pristane (SLE group) or saline (controls) as previously described (Satoh and Reeves, 1994). SLE mice were confirmed by the measurement of autoantibodies and IL-6 1 month after pristane injection; autoantibodies expressed at levels no less than the relative expression levels of control mice plus 3-fold of the SD, including autoantibodies against dsDNA (anti-dsDNA), Sm (anti-Sm), and histones (anti-histones), were chosen. Sixty SLE mice were randomly subdivided into groups and intragastrically administered 0.5% CMC-Na or 3, 10, or 30 mg/kg coptisine.

Coptisine was dissolved in 0.5% sodium carboxymethylcellulose (CMC-Na) to final concentrations of 0.15, 0.5, and 1.5 mg/mL. Beginning at 1.5 months, the mice were intragastrically administered 0.5% CMC-Na or coptisine for four and half months.

The mice were sacrificed 6 months after pristane injection. The mice were anaesthetized with chloral hydrate (400 mg/kg, i.p.). The wet spleen weight was measured and normalized to body weight. The thoracic aorta and kidneys were removed for further study, and blood and urine samples were collected and stored at -80°C.

**Figure S2** describes the experimental protocol.

### Behavioral Experiments

Spontaneous performance was tested using an autonomic activities tester equipped with eight individual darkrooms and infrared thermo probes. The BALB/c mice were placed in the independent darkroom in quiet conditions for 1 min to preadapt to the environment. During a following period of 5 min, times on spontaneous activity of each mouse was detected by the infrared probes.

### Preparation of Serum and Tissues

Serum was collected from the tail vein every month and kept at room temperature. Two hours later, the blood was centrifuged at 5,000 r·min^-1^ for 15 min at 4°C, and the supernatant was stored at -80°C to detect autoantibodies and cytokines.

Kidneys and spleens were immediately snap-frozen in liquid nitrogen and stored at -80°C. Spleens were fixed in 4% buffered paraformaldehyde.

### Histological and Immunofluorescence Analysis of Spleens

Spleen samples were fixed in 4% buffered paraformaldehyde and embedded in paraffin. Reactive oxygen species (ROS) levels in spleen cryosections were determined by 10 μm DHE fluorescence microtopography. Immunolocalization of activated macrophages was carried out by staining with a primary CD68 antibody. An anti-rabbit HRP/DAB detection system was used to visualize expression according to the manufacturer’s protocol. Sections were imaged under light or fluorescence light microscopy (Nikon, Japan) with a high-resolution camera.

### Blood Pressure

The systolic blood pressure (SBP), mean blood pressure (MBP), and diastolic blood pressure (DBP) of awake mice were measured using a Softron BP-98A tail-cuff haemodynamometer (Softron, Tokyo, Japan) every 2 months. Before measurement, mice were placed in the instrument in quiet condition for 5 min to preadapt to the environment.

### Assessment of Vascular Function

Assessment of vascular function, including vasoconstriction and endothelium-dependent and endothelium-independent vasorelaxation, was performed as previously reported (Dalaklioglu et al., 2013; Schmid et al., 2014). Briefly, following euthanasia, the thoracic aorta was excised and cleaned. The endothelium was intact, and the aorta was cut into strips (2 mm in length). The aortic rings were suspended by a pair of stainless pins in a well-oxygenated (95% O_2_, 5% CO_2_) bath containing K-H solution, pH 7.4, composed of 120 mm NaCl, 4.8 mm KCl, 1.2 mm KH_2_PO_4_, 25 mm NaHCO_3_, 11 mm glucose, 2.5 mm CaCl_2_, 1.4 mm MgCl_2_, and 0.01 mm ethylene diaminetetraacetic acid (EDTA) at 37°C and stabilized for 60 min under a resting tension of 1 g. During this time, the K-H solution was changed every 20 min during the equilibration period. The tension of the aortic rings was recorded isometrically with a force displacement transducer connected to a BIO-PAC polygraph (MP100A). First, studies to characterize the constrictive properties of the vessels were performed. Increasing concentrations of PE (10^-10^-10^-4^ M) were added to the bath to establish a contraction concentration-response curve. Then, endothelium-dependent and endothelium-independent vasorelaxation were assessed. After pre-contraction with PE (10^-6^ M), endothelium-dependent vasodilation was assessed with increasing concentrations of ACh (10^-10^-10^-5^ M), and endothelium-independent vasodilation was assessed with SNP (10^-10^-10^-6^ M).

### Detection of Autoantibodies

Autoantibodies were detected as previously described (Satoh et al., 1995a; Satoh et al., 1995b). ELISA plates (96 wells) were coated with 50 μL of 50 μg/mL calf thymus dsDNA, 0.5 μg/mL histone, or 0.5 μg/mL Sm at 4°C overnight. After washing three times with PBS containing 0.5% Tween-20 (PBST), the plates were blocked with 5% BSA in PBST for 2 h. Diluted serum was then added, and the plates were incubated for 3 h at room temperature. After washing, biotin conjugated horse anti-mouse IgG or goat anti-mouse IgM was incubated for 1 h at 37°C. After washing, HRP-conjugated streptavidin was incubated for 0.5 h at 37°C and washed again. TMB substrate was used to develop the colour, and the reaction was stopped with 2 N H_2_SO_4_. The absorbance was measured at 450 nm. The relative expression levels of the autoantibodies in each group were used to determine the mean enzyme index (EI), which was calculated as follows (Zhou et al., 2010):

EI=OD450 of the samplemean OD450 of the control + 3×SD×100%

### ELISA Analysis

Total serum levels of IgG, IL-6, IL-17A, monocyte chemoattractant protein-1 (MCP-1), intercellular adhesion molecule-1 (ICAM-1), renal tumour necrosis factor alpha (TNF-α), IL-6, IL-17A, and MCP-1 were measured using ELISA kits (eBioscience, San Diego, CA) according to the manufacturer’s instructions. The concentrations of the cytokines were quantified by reference to standard curves. The absorbance value of each renal sample was standardized to the individual protein concentration.

### Renal Injury

Urine protein was measured by a BCA protein assay kit. Urine creatinine was measured using a commercial kit (BioSino Bio-technology and Science Inc., Beijing, China). The data were expressed as milligrams of albumin per millimole creatinine.

For histopathology, kidneys were harvested from exsanguinated mice, immediately fixed in 4% buffered paraformaldehyde, and then embedded in paraffin. Renal sections (4 μm) stained with haematoxylin & eosin (H&E) and periodic acid-Schiff (PAS) were used for morphometric analysis of activity indices for lupus nephritis (LN). For immunofluorescence, tissues were snap-frozen in liquid nitrogen, sectioned and fixed in paraformaldehyde. The sections were stained with Texas Red 594-labelled donkey anti-goat IgG (1:100; Invitrogen, USA) at room temperature or rabbit anti-mouse C3 (1:100; Abcam, Cambridge, UK) and then incubated with Texas Red 594-labelled donkey anti-rabbit IgG (1:100; Invitrogen, USA). Nuclei were stained with DAPI (1:1,000; Invitrogen, USA). Sections were visualized with a Nikon Eclipse Ti-U fluorescence microscope (Nikon, Japan). The fluorescence intensity of the glomeruli from each animal was calculated using Image-Pro Plus v6.0.

### Western Blotting

Tissues were pulverized using a Bead Ruptor 24 with an Omni BR-Cryo Cooling Unit (Omni International, Georgia, USA) and extracted in radio immunoprecipitation assay (RIPA) lysis buffer containing 1 mm PMSF. The homogenates were incubated for 0.5 h on ice to achieve full lysis and then centrifuged at 10,000 r·min^-1^ for 10 min at 4°C. The supernatants were transferred in aliquots to new tubes. The soluble proteins were quantified by a BCA protein assay kit. After being mixed with loading buffer and boiled for 10 min, the lysates were subjected to SDS-PAGE and then transferred onto PVDF membranes (Millipore, Billerica, MA, USA). After blocking, the immunoblots were incubated with the primary antibodies overnight at 4°C. After incubation with an HRP-linked secondary antibody, the blots were individually visualized using an enhanced chemiluminescence reagent kit. The bands were quantified by Quantity One software (Bio-Rad, Richmond, CA, USA) and normalized to GAPDH as an internal control.

### Statistical Analysis

All data are expressed as the mean ± SEM. The significance of the differences between groups was determined by one-way ANOVA followed by Dunnett’s multiple comparison test. A *P* value less than 0.05 was regarded as significant. The images in this article were created using GraphPad Prism 5 (GraphPad Software Inc., La Jolla, CA, USA).

## Results

### Pristane-Induced Lupus

Facial hair loss was observed in 8 of the 80 BALB/c mice at 1 month after pristane injection (**Figure S3A**). Serum IgG and IgM autoantibodies against dsDNA, Sm, and histones, as well as total IgG, were detected every month. One month after pristane injection, these autoantibodies were significantly increased in SLE mice compared to control mice (**Figures S3B–H**). The level of IL-6 in SLE mice serum was 5-fold that of the level in control mice (**Figure S3I**).

### Safety of Coptisine in SLE Mice

During the 180-day administration of coptisine, no animal deaths or toxic symptoms were observed. The body weights of each group of mice showed no significant difference at 2 and 6 months after pristane injection (**Figure S4**).

As shown in **Figure S5**, significant decreases in spontaneous activities of SLE mice were observed at 2, 4, and 6 months after pristane injection. Administration of coptisine (30 mg/kg) significantly improved the activity of SLE mice at 4 months.

### Coptisine Reduces Autoantibody Production and the Expression of Cytokines in SLE Mice

**Figure 1:**
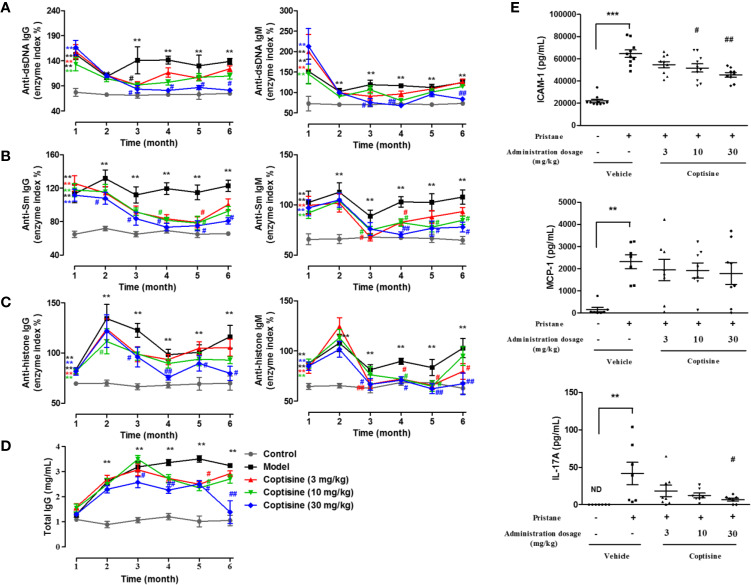
Coptisine attenuated autoantibody production and cytokine expression in SLE mice. Mice were injected with pristine (0.5 mL, i.p.) and then treated with coptisine (3, 10, or 30 mg/kg, i.g., q.d.) from 1.5 to 6 months. Sera were harvested from 1 to 6 months, and the levels of autoantibodies, such as IgG and IgM anti-dsDNA **(A)**, anti-Sm **(B)**, anti-histone **(C)** and total IgG **(D)**, were determined by ELISA (n=10-15). **(E)** The serum levels of the cytokines ICAM-1, IL-17A and MCP-1 were determined by ELISA at 6 months. The data are expressed as the mean ± SEM (n=7-10); **P < 0.01, ***P < 0.001 compared with the control group; ^#^*P* < 0.05, ^##^*P* < 0.01 compared with the model group.

In humans, SLE follows a relapsing/remitting course, and in SLE mice, there seems to be a similar phenomenon. IgG and IgM anti-dsDNA antibody levels decreased sharply at 2 months and then remained relatively steady; IgG and IgM anti-Sm antibody levels remained at a higher level from 1 to 6 months; IgG and IgM anti-histone antibody levels peaked at 2 months and then declined; the total IgG level increased gradually from 1 to 6 months after pristane injection. After treatment with coptisine for one and a half months, serum autoantibody levels in the SLE mice were dramatically continuously restored to normal levels (**Figures 1A–D**). Abnormalities in cytokines, as important key players in the immune system, have been implicated in the pathogenesis of SLE, either as part of the pathogenetic core process of lupus or as secondary markers indicating immune dysregulation (Apostolidis et al., 2011). We detected serum cytokines, including ICAM-1, IL-17A, and MCP-1. At 6 months, serum cytokine levels were elevated in SLE mice compared with the control group, while coptisine significantly reduced the expression of ICAM-1 and IL-17A (**Figure 1E**).

### Coptisine Improves Spleen Pathologic Characteristics

**Figure 2:**
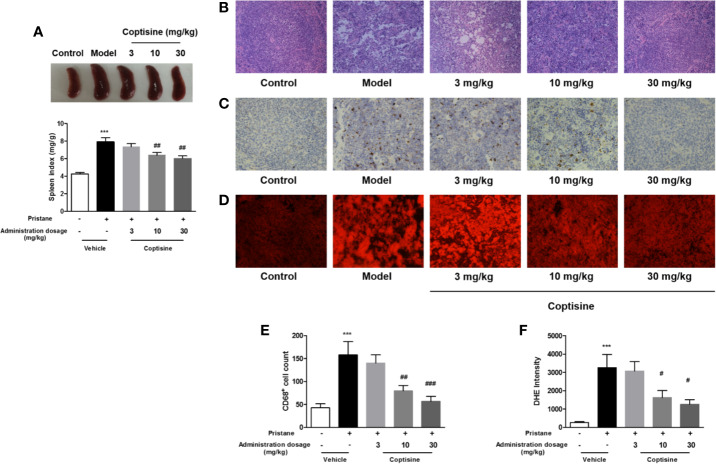
Coptisine decreased the spleen index and ROS and macrophage accumulation in the spleen. **(A)** The level of splenomegaly is represented as the spleen index and is expressed as milligrams of spleen per kilograms of body weight (n=15). **(B)** Pathological changes in the spleen were assessed by H&E staining. **(C)** Representative immunohistochemical staining for CD68 (All images are 200×). **(D)** Oxidative stress in the spleen was examined with DHE fluorescent dye (All images are 200×). **(E, F)** Quantitative analysis of CD68 and ROS levels using 16 images collected from 4 mice. The data are expressed as the mean ± SEM; ***P < 0.001 compared with the control group; ^#^*P* < 0.05, ^##^*P* < 0.01, ^###^*P* < 0.001 compared with the model group.

The spleen, an important lymph organ in the body, plays a role in immunity; meanwhile, SLE causes splenomegaly as a result of disordered immune regulation. We assessed the level of splenomegaly by testing the spleen index, expressed as milligrams of spleen per kilograms of body weight. Compared to control mice, SLE mice showed severe splenomegaly, while treatment with coptisine (10 and 30 mg/kg) significantly decreased the spleen index (**Figure 2A**). Spleen histopathology analysis revealed that the development of the germinal centre (GC) was indicated by the lighter areas in the centre of the white pulp in the SLE group, while spleens from control mice displayed fewer and smaller white pulp areas (**Figure 2B**). The fluorescence intensity of DHE, a cell-permeable probe that indicates the formation of ROS, and staining for CD68, a marker of monocytes/macrophages, were significantly higher in spleens from SLE mice compared with those from control animals. Treatment with coptisine (30 mg/kg) reduced both the DHE fluorescence intensity and CD68 level (**Figures 2C–F**).

### Coptisine Improves Renal Injury

**Figure 3:**
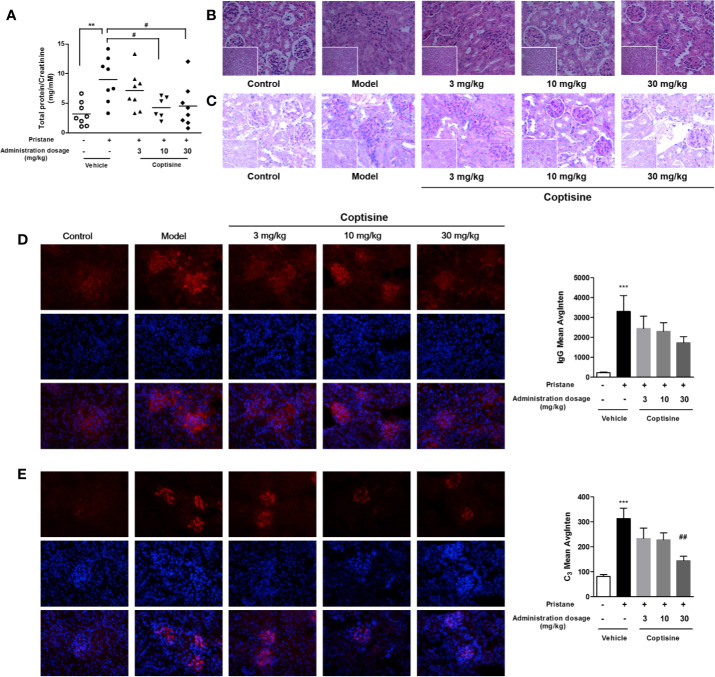
Coptisine ameliorated SLE-induced renal dysfunction. **(A)** The proteinuria level is expressed as urinary protein excreted relative to urinary creatinine excreted (n=8). **(B, C)** Pathological changes in the kidney were assessed by H&E staining **(B)** and PAS staining **(C)** (n=5, the small images are 200×, the big images are 400×). **(D, E)** Left, representative images of the deposition of IgG **(D)** and C3 **(E)** in the glomeruli, as tested by immunofluorescence. The top row shows IgG **(D)** and C3 **(E)**, the middle row shows DAPI, and the bottom row is an overlay of the two. IgG was stained with red fluorescent dye (red; exposure time, 100 milliseconds), C3 was stained with red fluorescent dye (red; exposure time, 200 milliseconds), and the nuclei were stained with DAPI (blue; exposure time, 30 milliseconds). Right, quantitative analysis of IgG and C3 levels using 9 images collected from 3 mice. The data are expressed as the mean ± SEM; **P < 0.01, ***P < 0.001 compared with the control group; ^#^*P* < 0.05, ^##^*P* < 0.01 compared with the model group.

Proteinuria was strikingly increased in SLE mice compared with control mice, and treatment with coptisine (10 or 30 mg/kg) reduced proteinuria levels in SLE mice to 4.26 and 4.49 μg/μm, respectively [vs. the vehicle SLE group (9.00 μg/μm)] (**Figure 3A**). Kidney H&E staining (**Figure 3B**) showed that SLE induced severe kidney injury in mice, including glomerular hypercellularity and tubular epithelial cell swelling and necrosis. The tissue infiltration of leucocytes and glomerulonephritis in SLE mice was higher than that in control mice. Renal damage was less severe in mice treated with coptisine (10 or 30 mg/kg) (**Figure 3B**). In glomeruli, as shown in **Figure 3C**, the PAS-stained area, indicative of mesangial matrix expansion and glomerulosclerosis, was increased in SLE mice compared with control mice. In addition, epithelial and endothelial cells were hypertrophic, and capillary loops were thickened; however, these changes were not apparent in the glomeruli of control mice. SLE mice treated with coptisine (30 mg/kg) exhibited a decreased area of sclerosis and ameliorated mesangial matrix expansion (**Figure 3C**). Immunofluorescence studies reinforced the morphologic findings of more pronounced scattered granular deposits of IgG (**Figure 3D**) and C3 (**Figure 3E**) in the glomeruli of vehicle-treated SLE mice than control mice. SLE mice treated with coptisine (30 mg/kg) showed remarkably diminished C3 deposition (**Figure 3E**) and a trend of weakened IgG deposition (**Figure 3D**).

### Coptisine Partially Decreases Inflammatory Responses in the Kidney

**Figure 4:**
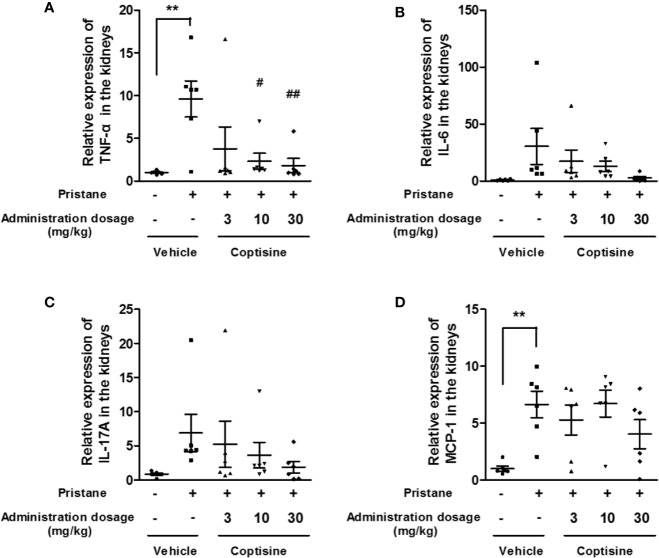
Coptisine partially decreased inflammatory responses in the kidney. The levels of TNF-α **(A)**, IL-6 **(B)**, IL-17A **(C)**, and MCP-1 **(D)** in the kidney were measured by ELISA (n=6). The data are expressed as the mean ± SEM; **P < 0.01 compared with the control group; ^#^*P* < 0.05, ^##^*P* < 0.01 compared with the model group.

To determine whether coptisine treatment altered renal inflammation, we assessed the renal protein expression of TNF-α, IL-6, IL-17A, and MCP-1. At 6 months, the expression levels of TNF-α and MCP-1 in the kidneys were increased in SLE mice compared with control mice (**Figure 4**). Coptisine (10, 30 mg/kg) significantly attenuated the increase of TNF-α (**Figure 4A**), however, coptisine partially alleviated this trend of MCP-1 without significant difference (**Figure 4D**).

### Coptisine Partly Inhibits Rho/ROCK Pathway in the Kidney

**Figure 5:**
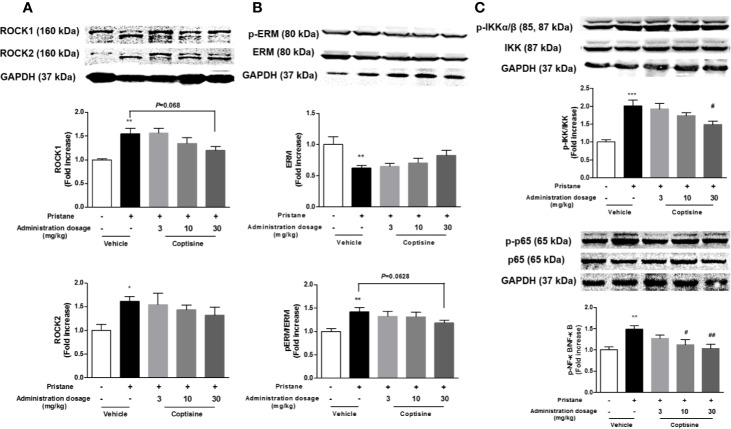
Coptisine exhibited a trend of activity inhibition of Rho/ROCK pathway in the kidney. **(A)** Representative immunoblotting bands of ROCK1 and ROCK2. **(B)** The phosphorylation levels of ERM, a downstream target of ROCKs. **(C)** The activity of canonical NF-κB pathway were examined by western blotting. The data are expressed as the mean ± SEM (n=6); *P < 0.05, **P < 0.01 compared with the control group; ^#^*P* < 0.05, ^##^*P* < 0.01 compared with the model group.

To explore the molecular mechanisms involved in coptisine-mediated kidney protection, we further investigated the effects of coptisine on the Rho/ROCK pathway. As shown in **Figures 5A, B**, western blotting indicated that Rho/ROCK signalling pathway was significantly activated in SLE mouse kidneys, coptisine (30 mg/kg) exhibited a trend of activity inhibition.

RhoA/ROCK signalling is involved in the production of pro-inflammatory cytokines through the regulation of NF-κB activation. As shown in **Figure 5C**, NF-κB activation in the kidneys were increased in SLE mice compared with control mice, and coptisine (30 mg/kg) significantly alleviated this trend.

### Coptisine Improves Vascular Function

**Figure 6:**
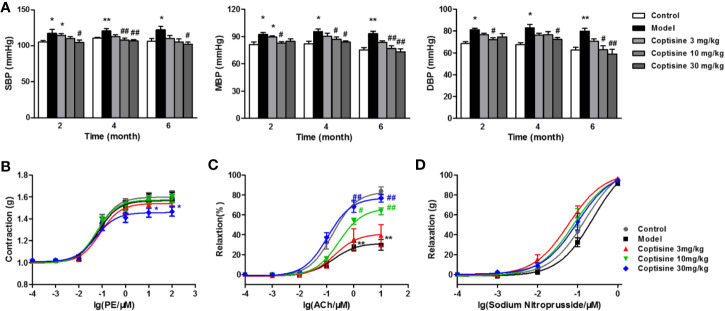
Coptisine reduced blood pressure and improved vascular function. **(A)** SBP, MBP, and DBP of awake mice were measured using a tail-cuff haemodynamometer at 2, 4, and 6 months (n=10-12). The concentration-response contraction of PE **(B)**, the endothelium-dependent vasodilatory response of ACh **(C)** and the endothelium-independent vasodilatory response of SNP **(D)** were measured in mouse aortic rings (n=6). The data are expressed as the mean ± SEM; *P < 0.05, **P < 0.01 compared with the control group; ^#^*P* < 0.05, ^##^*P* < 0.01 compared with the model group.

Blood pressure, including SBP, MBP, and DBP, increased in SLE mice compared to the control group beginning at 2 months, and coptisine (10 or 30 mg/kg) treatment significantly reduced blood pressure (**Figure 6A**).

The concentration-response contraction of PE (10^-10^-10^-4^ M) was not significantly different in the SLE group compared with the control group, while treatment with coptisine (30 mg/kg) dramatically decreased the maximal contraction to approximately 22% (**Figure 6B**). We tested the vasodilatory response of aortic rings to increasing concentrations of ACh (10^-10^-10^-5^ M) after pre-contraction with PE (10^-6^ M). ACh led to endothelium-dependent vasodilation by stimulating the production of NO in endothelial cells (ECs) and then relaxation of vascular smooth muscle cells (VSMCs). The maximal relaxation of SLE mouse aortic rings, which was reached at an ACh concentration of 10^-5^ M, was attenuated to 29.70% [vs. the vehicle control group (84.01%)], while coptisine (10 or 30 mg/kg) improved endothelial dysfunction by increasing the maximal responses to 63.69 and 76.88%, respectively (**Figure 6C**). SNP is a direct NO donor, so the observed vasodilation depended on VSMC function but not on endothelial function. As a result, the concentration-response curves of SNP (10^-10^-10^-6^ M) showed no significant differences between each group (**Figure 6D**).

## Discussion

SLE is a complex and potentially life-threatening autoimmune disease characterized primarily by the production of autoantibodies against self-antigens, and it affects 1.5 million Americans and at least five million people worldwide (Kinsey et al., 2018; Stojan and Petri, 2018). Therefore, new drugs still need to be identified. In this study, we established a pristane-induced lupus mouse model to evaluate the therapeutic effects of coptisine on SLE and renal and cardiovascular complications.

Pristane can induce systemic lupus with characteristic organ involvement and autoantibodies in various mouse strains. The intraperitoneal injection of pristane stimulates the formation of lupus-associated autoantibodies against multiple nuclear antigens. This leads to chronic inflammation with the development of lupus-like autoimmunity, which particularly includes the formation of antibodies characteristic of SLE as well as immune-complex nephritis with a high degree of similarity to human SLE (Freitas et al., 2017; Li et al., 2017). In our study, the mice showed lupus-like characteristics, including increased levels of autoantibodies, splenomegaly, and LN. For the first time, we found that this mouse model exhibits some cardiovascular complications, such as hypertension-like symptoms and vascular endothelial dysfunction.

ROCKs are emerging as important regulators of T cell effector function *via* their ability to be activated under Th17 conditions and to regulate the production of IL-17 and IL-21, and initial studies have revealed that ~60% of SLE patients exhibit increased ROCK activity in their PBMCs (Isgro et al., 2013). In SLE and RA patients, Th17 cells exhibit high ROCK activity that is inhibited by Y276327, KD025, or simvastatin; IL-17 and IL-21 production is also decreased by purified SLE T cells or Th17 cells (Rozo et al., 2017). In a previous study, coptisine was shown to alter RhoA and ROCK expression and inhibit the activity of ROCKs (Gong et al., 2012b; Guo et al., 2013); therefore, we hypothesized that coptisine might be effective in slowing the progression of SLE. In pristane-induced lupus mice, we found that coptisine treatment dramatically decreased the levels of serum autoantibodies, including IgG and IgM autoantibodies against dsDNA, Sm, histones, total IgG, and serum cytokines and improved the pathologic characteristics of the spleen, which indicated that coptisine had an ameliorative effect on the disease.

Renal involvement represents a major cause of morbidity and mortality, and LN remains a major therapeutic challenge (Cecchi et al., 2019). Kidney biopsies of patients with LN have shown that the activity of ROCKs increases in T cells, leading to ERM phosphorylation and CD44 overexpression, which are related to uropod size, cell migration and the construction of polar caps in lymphocyte cells (Li et al., 2007). On the other hand, renal injury in SLE is known to be mediated by immune complex deposition and downstream inflammation, and RhoA/ROCK signalling is involved in the production of pro-inflammatory cytokines through the regulation of NF-κB activation. In our study, lupus mice treated with coptisine showed significant improvement of renal function and structure, which was reflected by reductions in the rate of urinary protein to creatinine, glomerular size, the extent of mesangial matrix expansion and glomerulosclerosis, the deposition of IgG and C3 and the expression of pro-inflammatory cytokines. Western blot data demonstrated that coptisine administration partially downregulated RhoA/ROCK activity in the kidneys of pristane-induced SLE mice.

Another primary cause of mortality in SLE, particularly in women who survive beyond the first 5 years, is cardiovascular disease (Manzi et al., 1997). Numerous studies have reported that hypertension is common among patients with SLE, and studies have shown that it is more prevalent in SLE patients than in people without SLE (Munguia-Realpozo et al., 2019). In this study, blood pressure was monitored during the experiment, and the data showed that coptisine reduced blood pressure. Although the mechanisms of hypertension in SLE have not been elucidated, the importance of the kidneys in the long-term control of blood pressure and the pathogenesis of hypertension is well documented (Coleman et al., 1975). Therefore, it is not surprising that impaired renal function is a cause of the progression of SLE hypertension. Coptisine might reduce blood pressure by improving renal function. On the other hand, coptisine also shows an *in vitro* vasorelaxant effect on rat aortic rings (Gong et al., 2012a). Further study confirmed that the *in vivo* data were in accordance with the vasorelaxant effect of coptisine on the concentration-response contraction of PE in the aortic rings of the SLE. However, the vasodilatory mechanism of coptisine needs to be further confirmed.

SLE is an inflammatory autoimmune condition associated with endothelial dysfunction and elevates the risk of cardiovascular disease in young women by 50-fold (Sciatti et al., 2019). Treatment with coptisine significantly alleviated the impairment of the vascular relaxation response to the endothelium-dependent vasodilator ACh in SLE mice, which suggests that coptisine may prevent vascular endothelial dysfunction. The mechanisms of endothelial dysfunction in SLE patients may be upregulated expression of ICAM-1 and vascular cell adhesion molecule-1 (VCAM-1), increased secretion of chemokines such as MCP-1, IL-6, IL-17A, and TNF-α, and the promotion of the activation of the transcription factor NF-κB p65 in human endothelial cells by immune complexes (Gómez-Guzmán et al., 2014; Tan et al., 2014). In this study, we found that coptisine significantly alleviated the overexpression of ICAM-1 and IL-17A in the serum.

In conclusion, our findings indicate that coptisine slows disease progression in pristane-induced lupus mice by decreasing serum autoantibody levels and improving pathologic characteristics of the spleen. Furthermore, coptisine slows SLE-associated kidney and vascular complications. The protective effects of coptisine in pristane-induced lupus mice might be partially due to the inhibition of the Rho/ROCK pathway. Taken together, these findings suggest that therapy with coptisine may be a strategy for preventing SLE and ameliorating associated kidney and cardiovascular complications.

## Data Availability Statement

All datasets generated for this study are included in the article/**Supplementary Material**.

## Ethics Statement

The animal study was reviewed and approved by the Laboratories Institutional Animal Care and Use Committee of the Chinese Academy of Medical Sciences and Peking Union Medical College.

## Author Contributions

LF and GD conceived and designed the study. YY draft the manuscript. ZZ and HQ synthesized the compound and participated in draft the manuscript. YY and BH carried out the animal experiments and signaling studies. YC and KL carried out the statistical analysis.

## Funding

This study was supported by grants from the National Natural Science Foundation of China (nos. 81773935 and 81872761) and China-Japan Friendship Hospital Youth Science and Technology Excellence Project (nos. 2018-1-QN-15).

## Conflict of Interest

The authors declare that the research was conducted in the absence of any commercial or financial relationships that could be construed as a potential conflict of interest.
