# Supplementary figures and images for: Coptisine Alleviates Pristane-Induced Lupus-Like Disease and Associated Kidney and Cardiovascular Complications in Mice
Source: Front Pharmacol. 2020 Jun 19;11:929. doi: 10.3389/fphar.2020.00929 (PMC7316987; doi:10.3389/fphar.2020.00929)

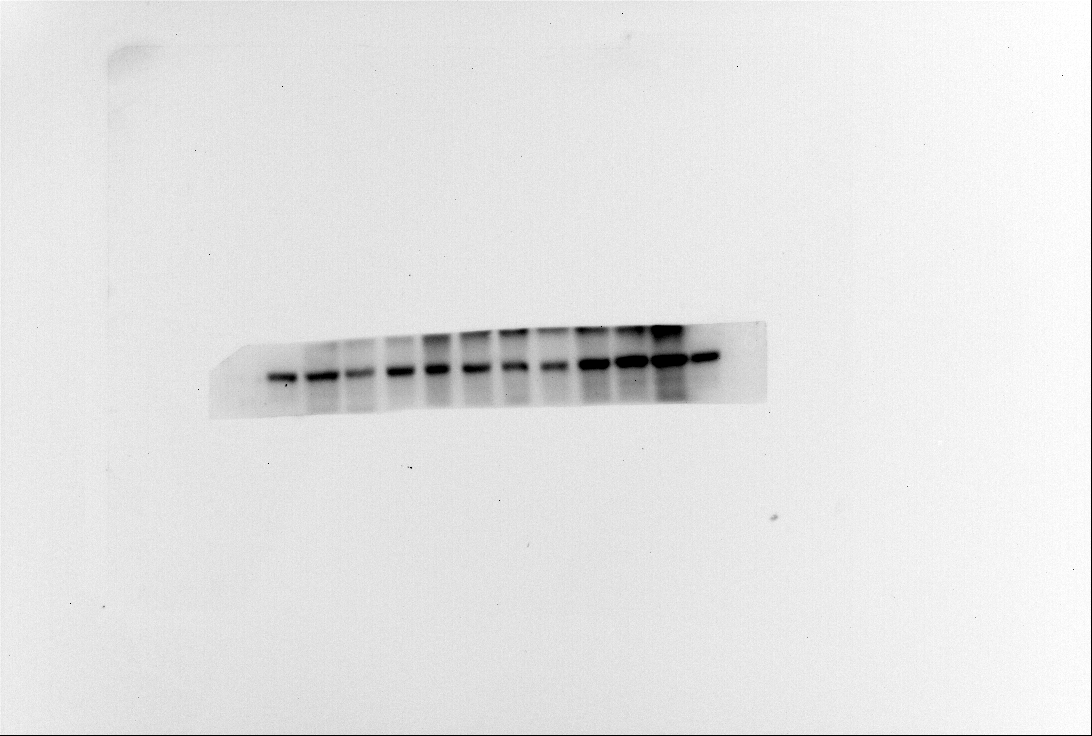

Supplement: Supplementary file 1 [file DataSheet_1.zip › original blots figures/original blots figures/5A/GAPDH.TIF]

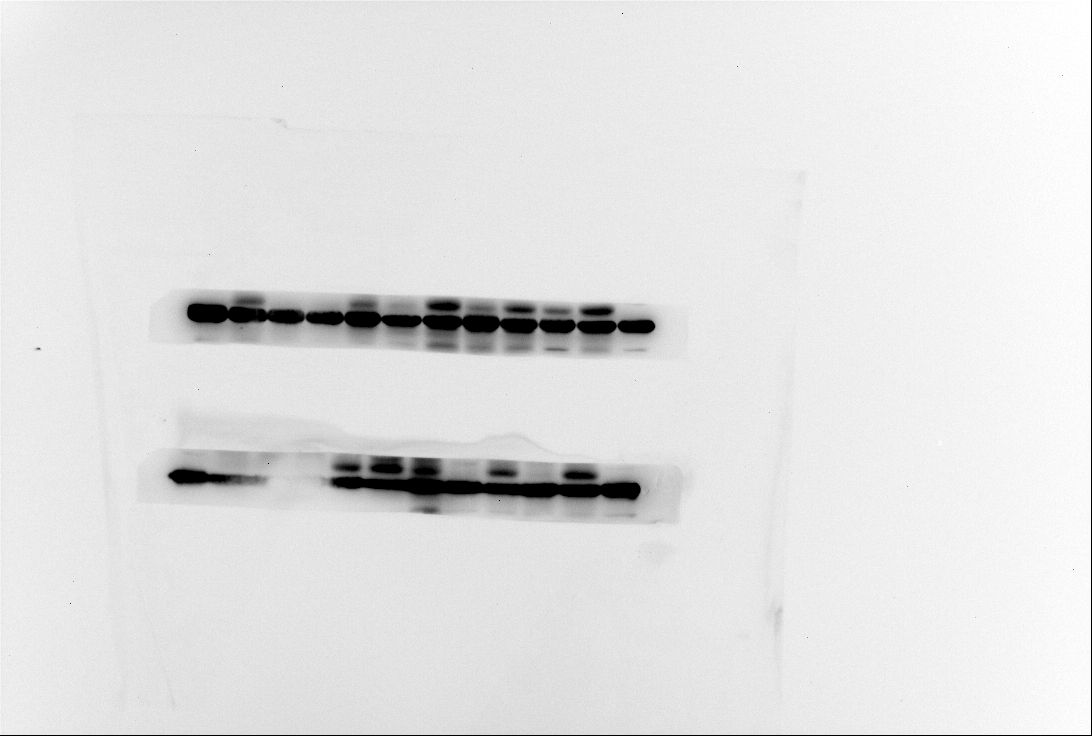

Supplement: Supplementary file 1 [file DataSheet_1.zip › original blots figures/original blots figures/5A/GAPDH-3.TIF]

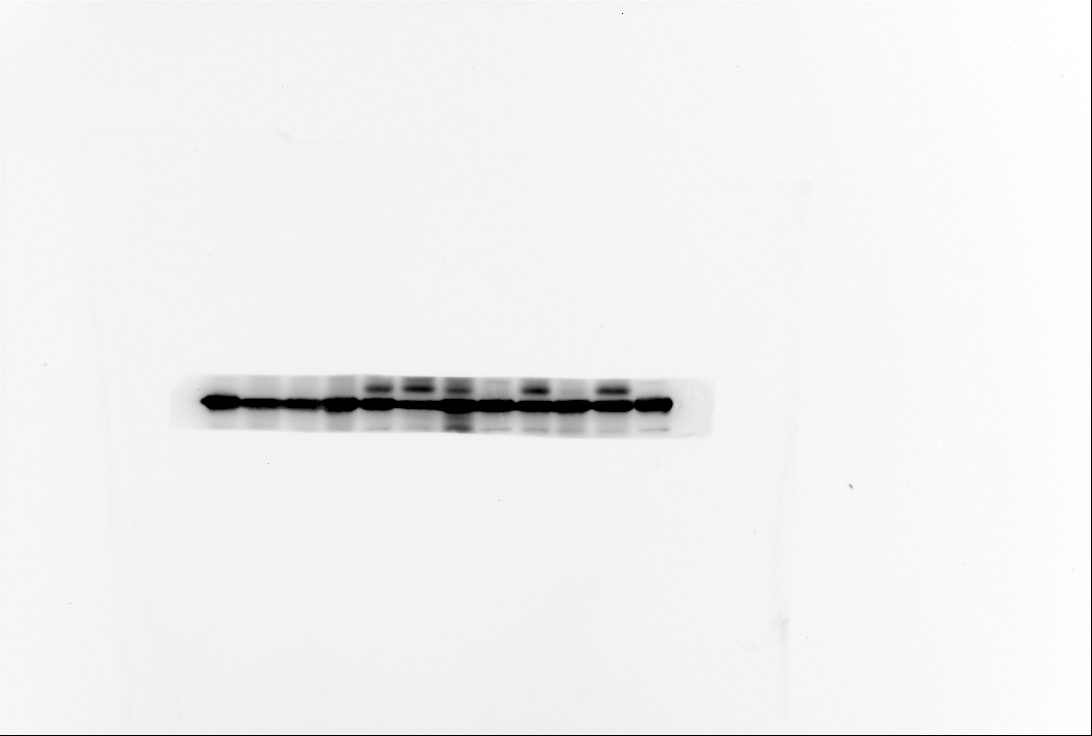

Supplement: Supplementary file 1 [file DataSheet_1.zip › original blots figures/original blots figures/5A/GAPDH-4.TIF]

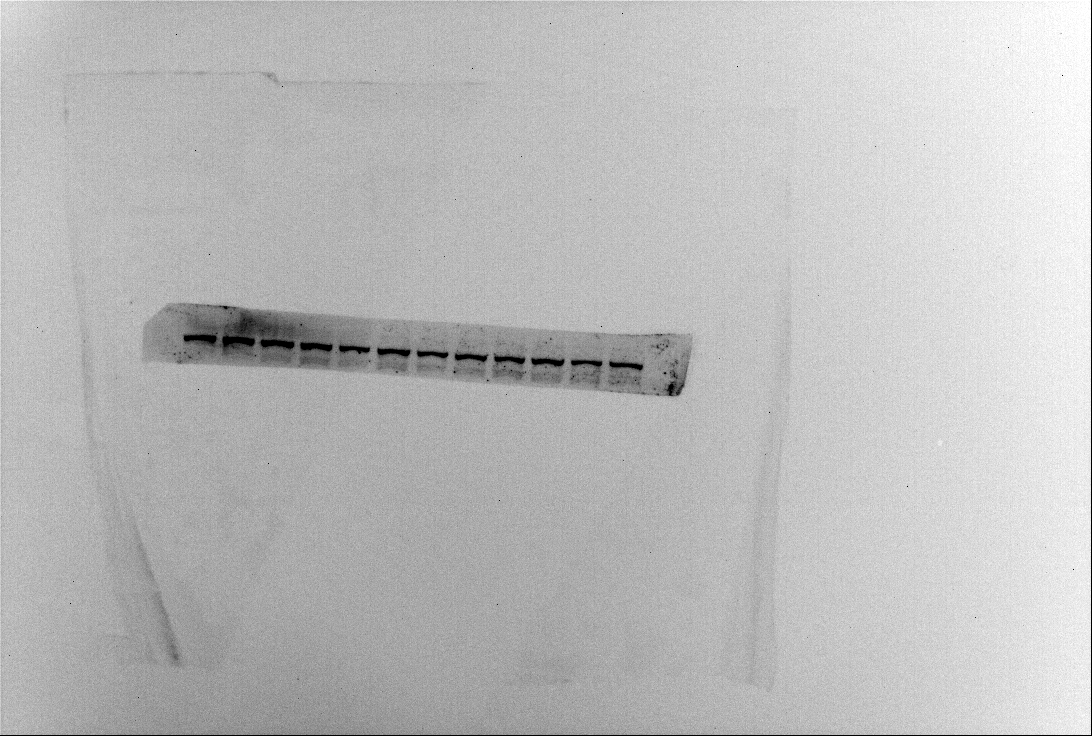

Supplement: Supplementary file 1 [file DataSheet_1.zip › original blots figures/original blots figures/5A/ROCK1-1.TIF]

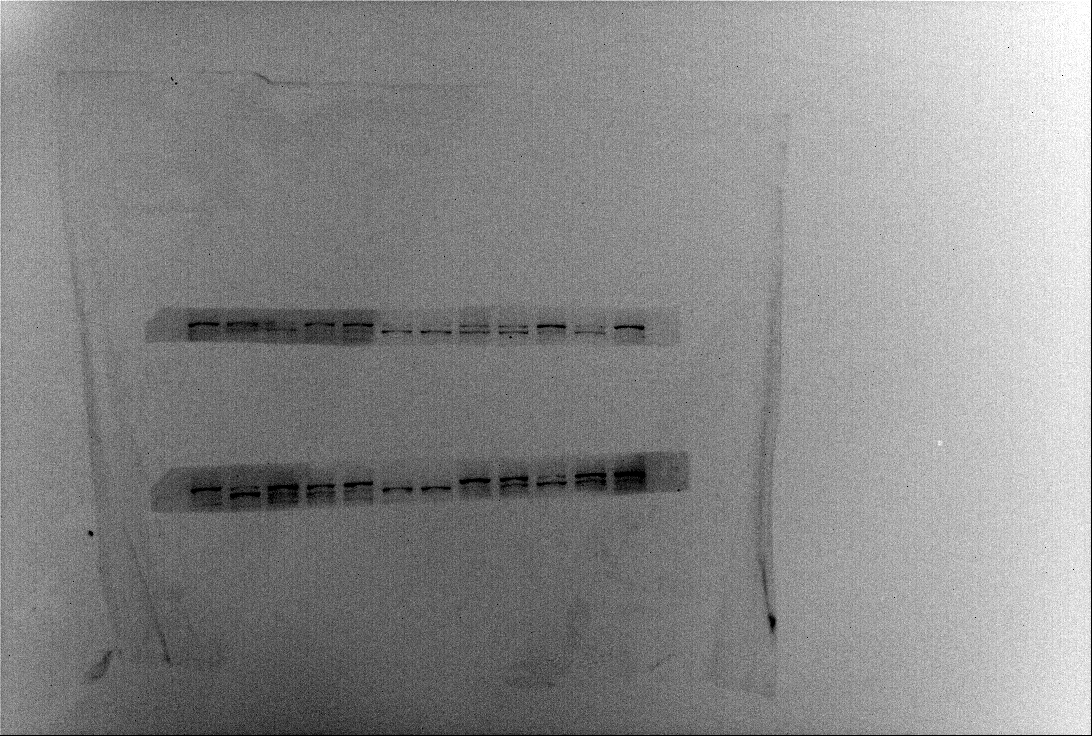

Supplement: Supplementary file 1 [file DataSheet_1.zip › original blots figures/original blots figures/5A/ROCK1-3_4.TIF]

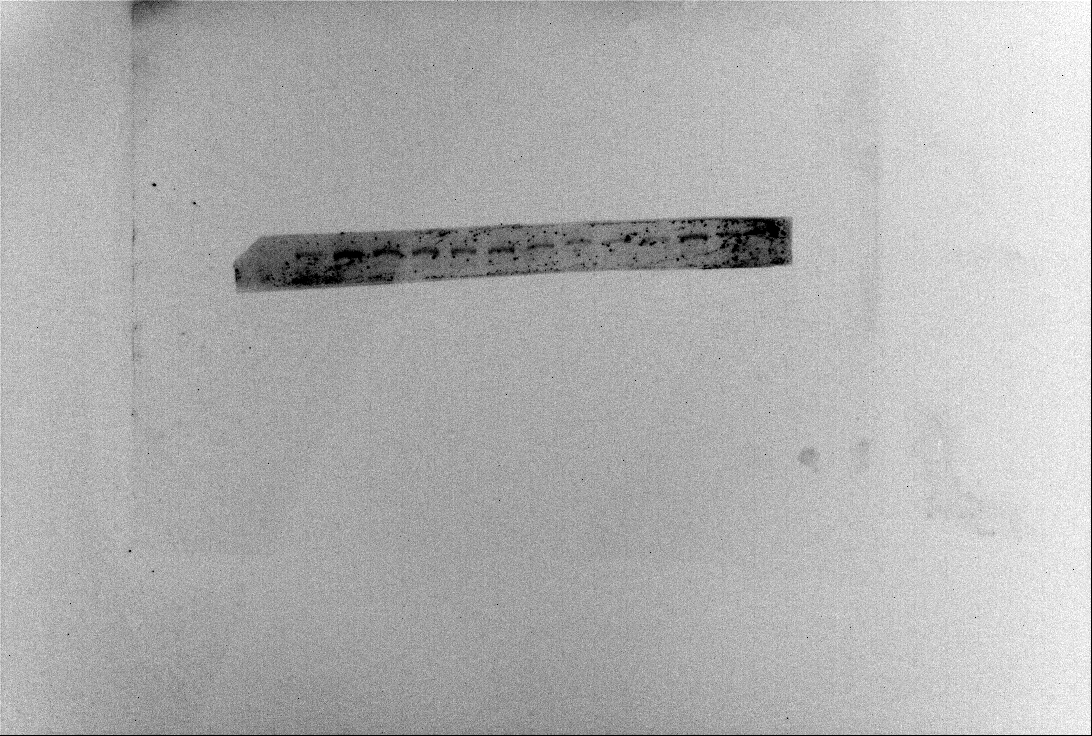

Supplement: Supplementary file 1 [file DataSheet_1.zip › original blots figures/original blots figures/5A/ROCK2-1.TIF]

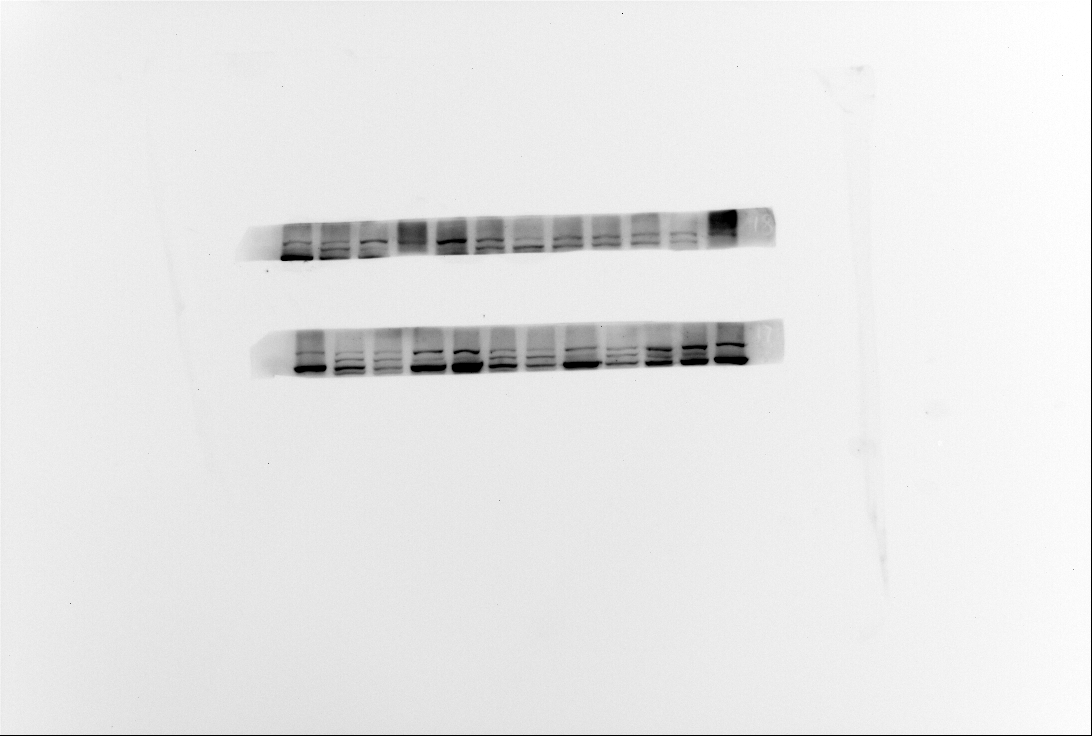

Supplement: Supplementary file 1 [file DataSheet_1.zip › original blots figures/original blots figures/5A/ROCK2-3_4.TIF]

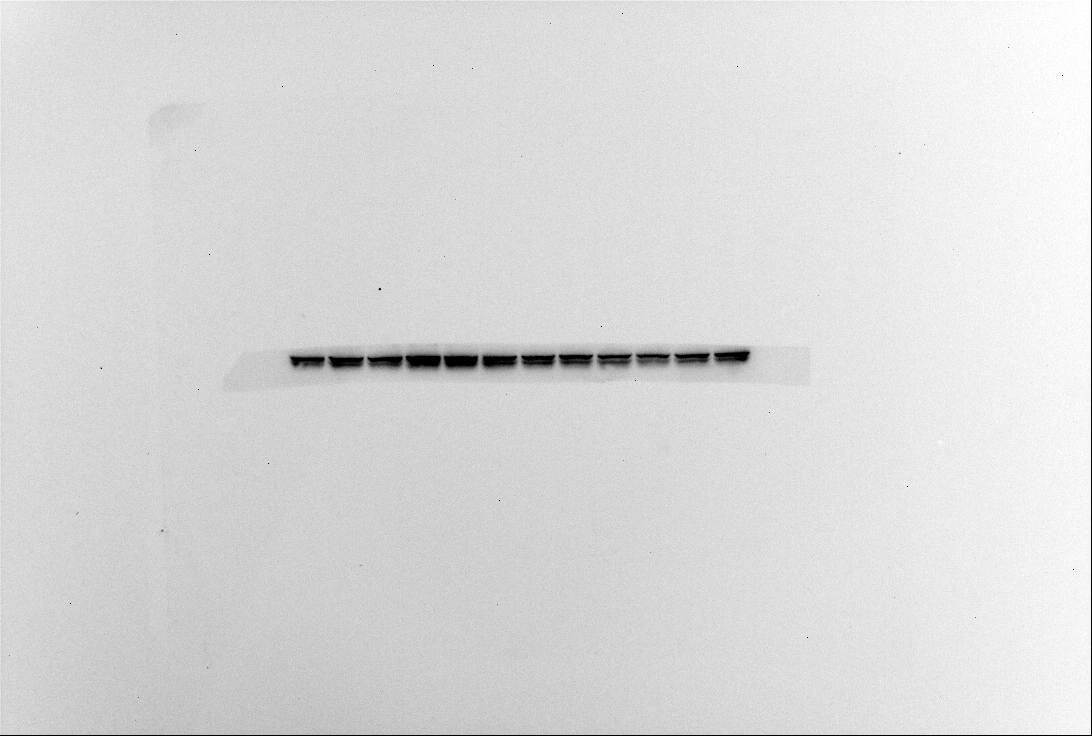

Supplement: Supplementary file 1 [file DataSheet_1.zip › original blots figures/original blots figures/5B/ERM.TIF]

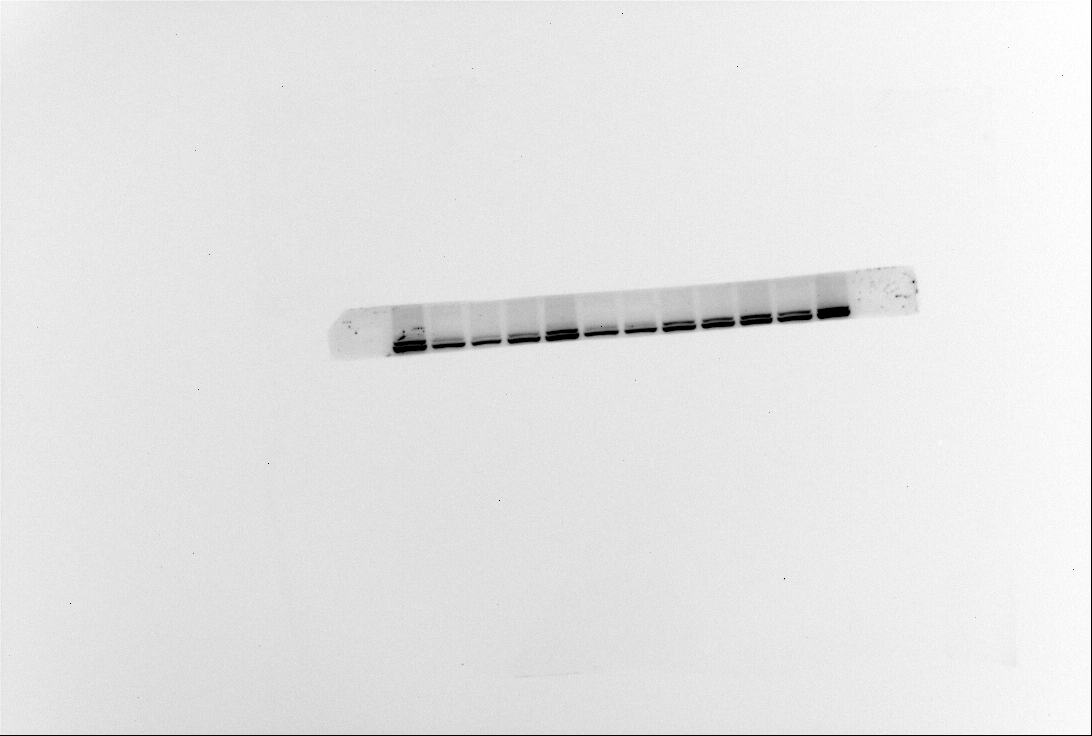

Supplement: Supplementary file 1 [file DataSheet_1.zip › original blots figures/original blots figures/5B/ERM-2.TIF]

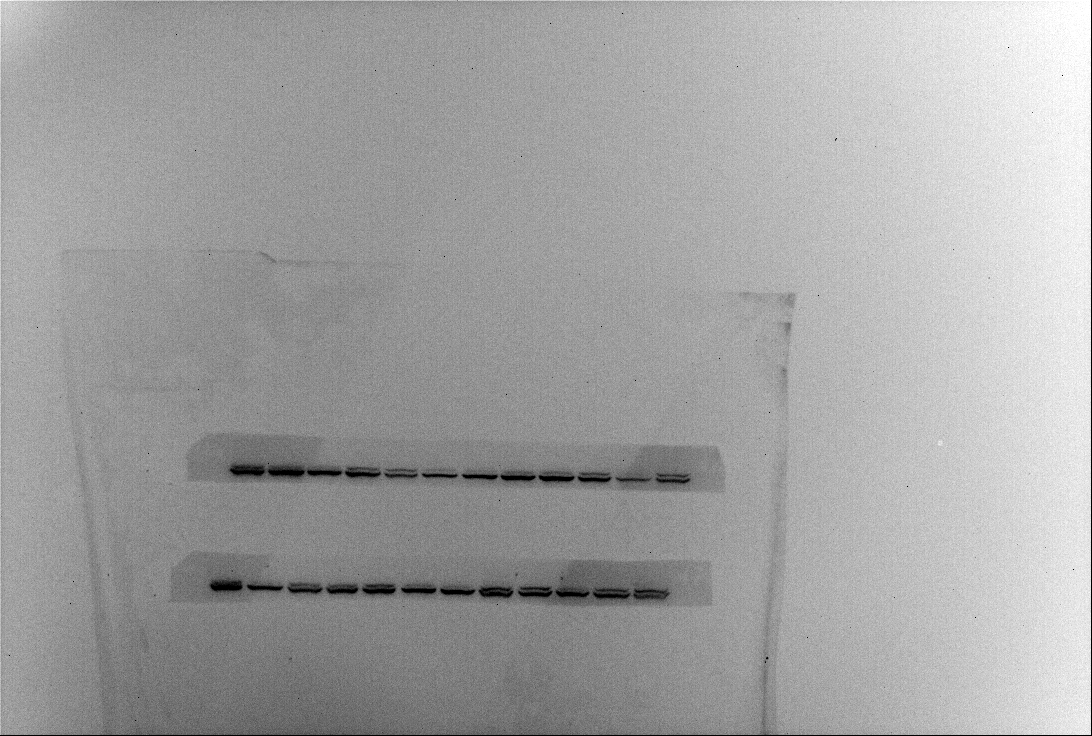

Supplement: Supplementary file 1 [file DataSheet_1.zip › original blots figures/original blots figures/5B/ERM-34.TIF]

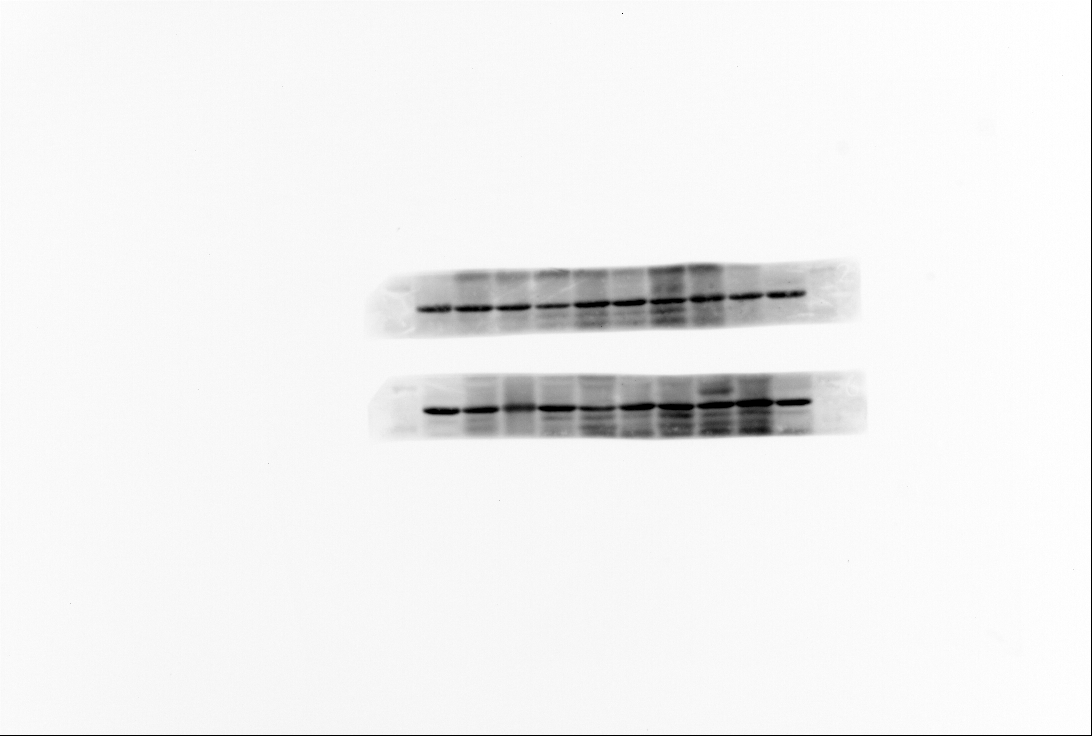

Supplement: Supplementary file 1 [file DataSheet_1.zip › original blots figures/original blots figures/5B/gapdh-1,2.TIF]

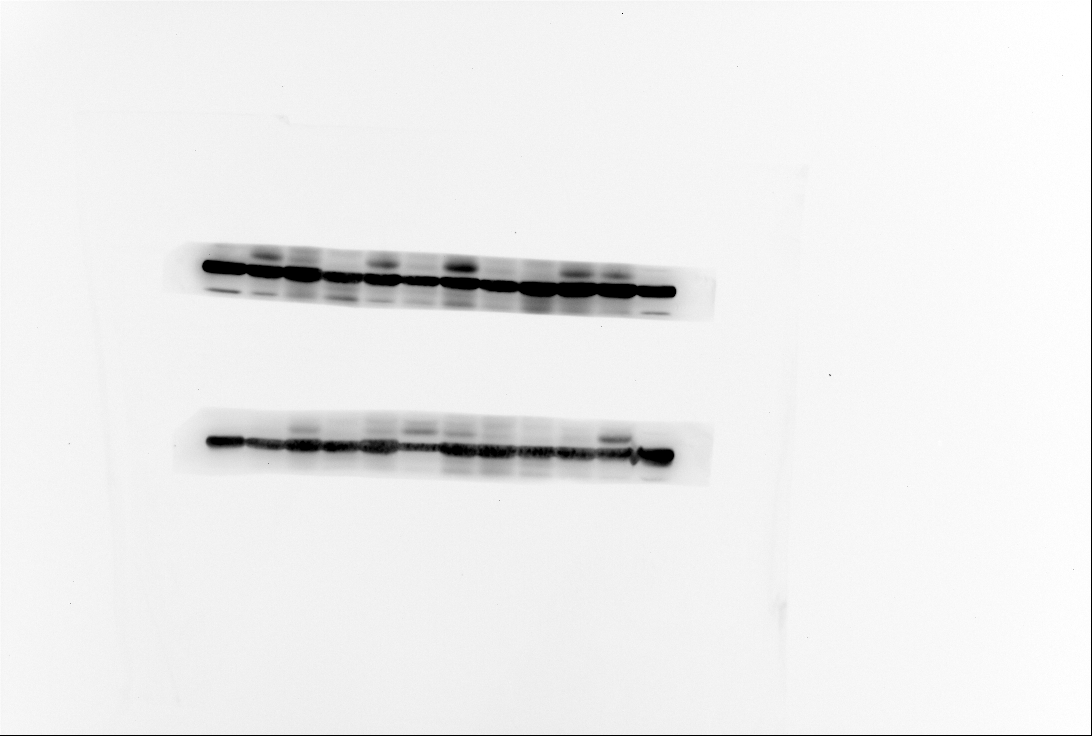

Supplement: Supplementary file 1 [file DataSheet_1.zip › original blots figures/original blots figures/5B/GAPDH-34.TIF]

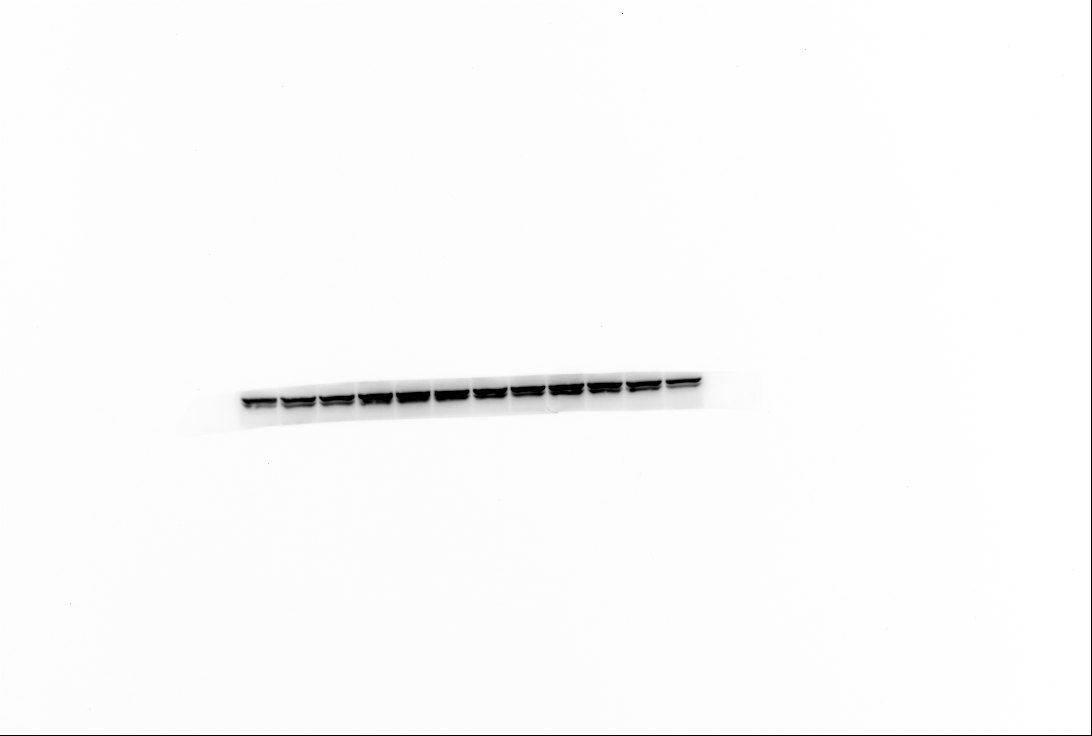

Supplement: Supplementary file 1 [file DataSheet_1.zip › original blots figures/original blots figures/5B/p-ERM.TIF]

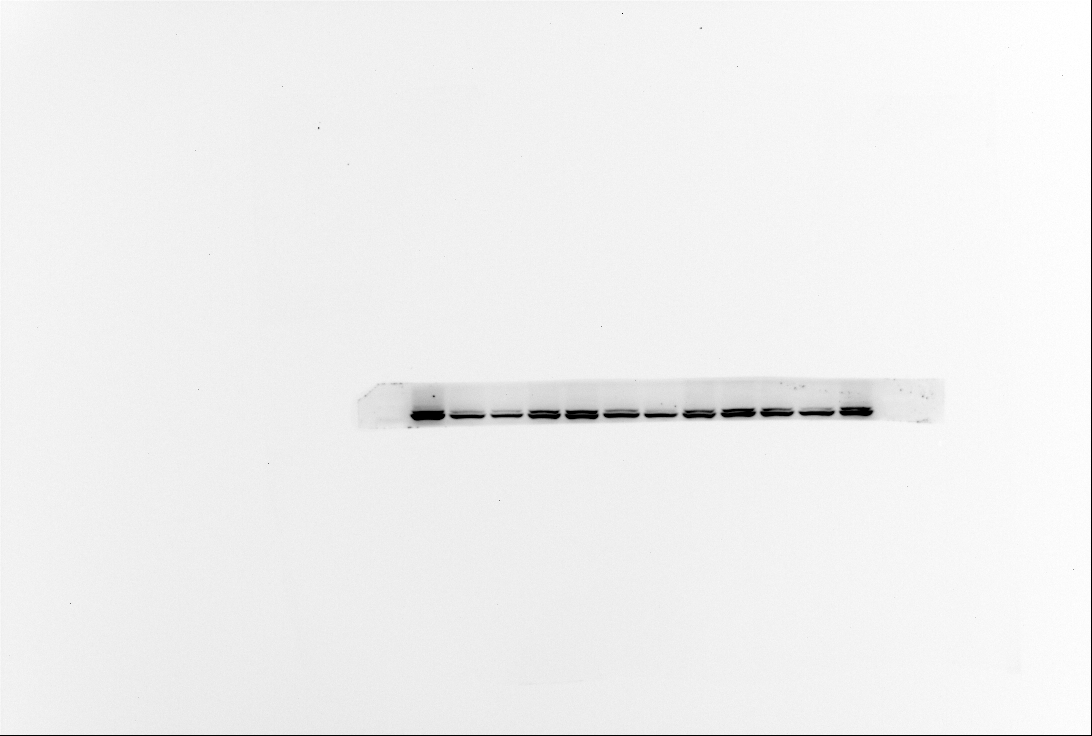

Supplement: Supplementary file 1 [file DataSheet_1.zip › original blots figures/original blots figures/5B/p-ERM-2.TIF]

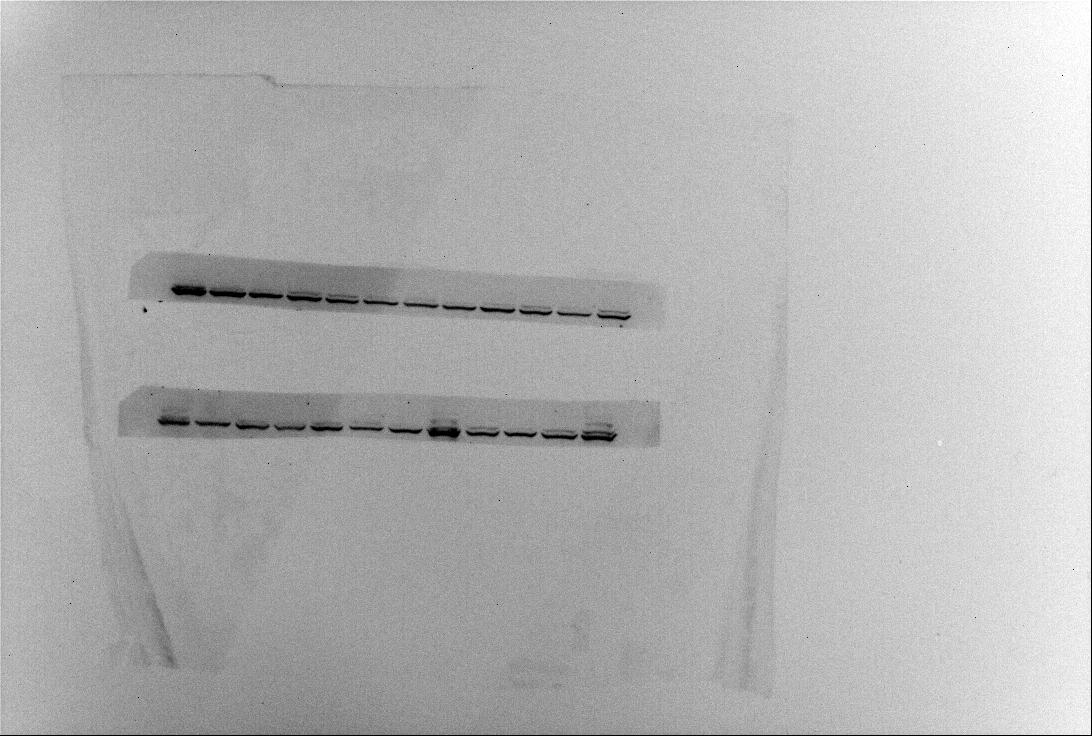

Supplement: Supplementary file 1 [file DataSheet_1.zip › original blots figures/original blots figures/5B/p-ERM-34.TIF]

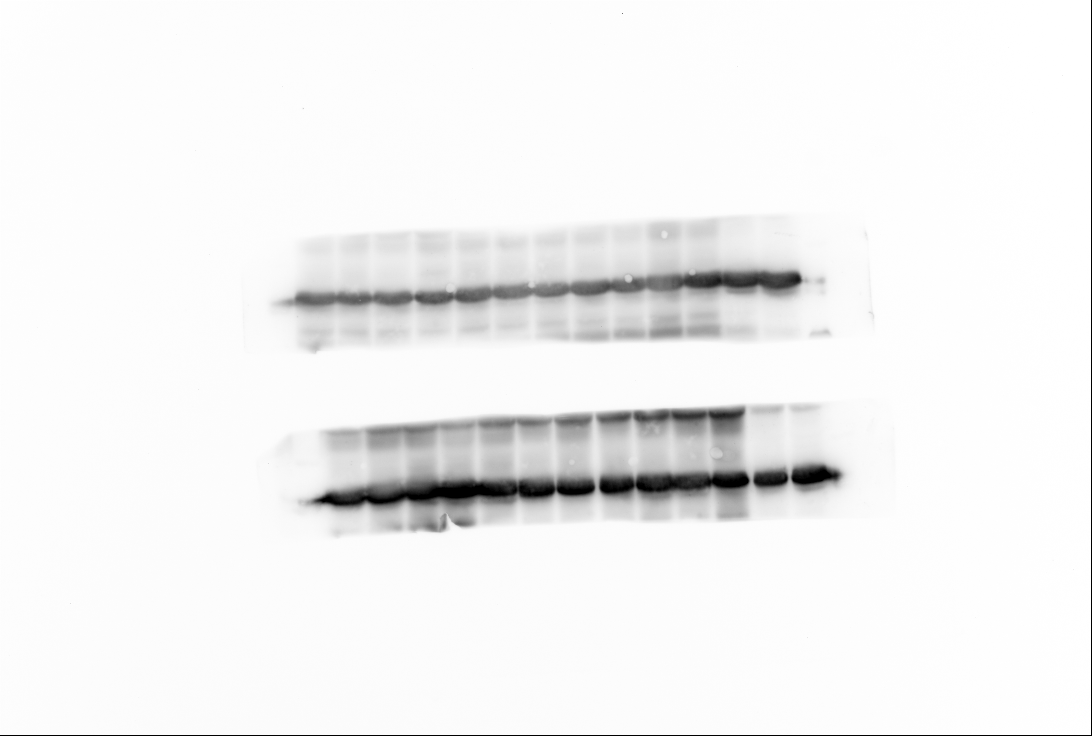

Supplement: Supplementary file 1 [file DataSheet_1.zip › original blots figures/original blots figures/5C/GAPDH-12.TIF]

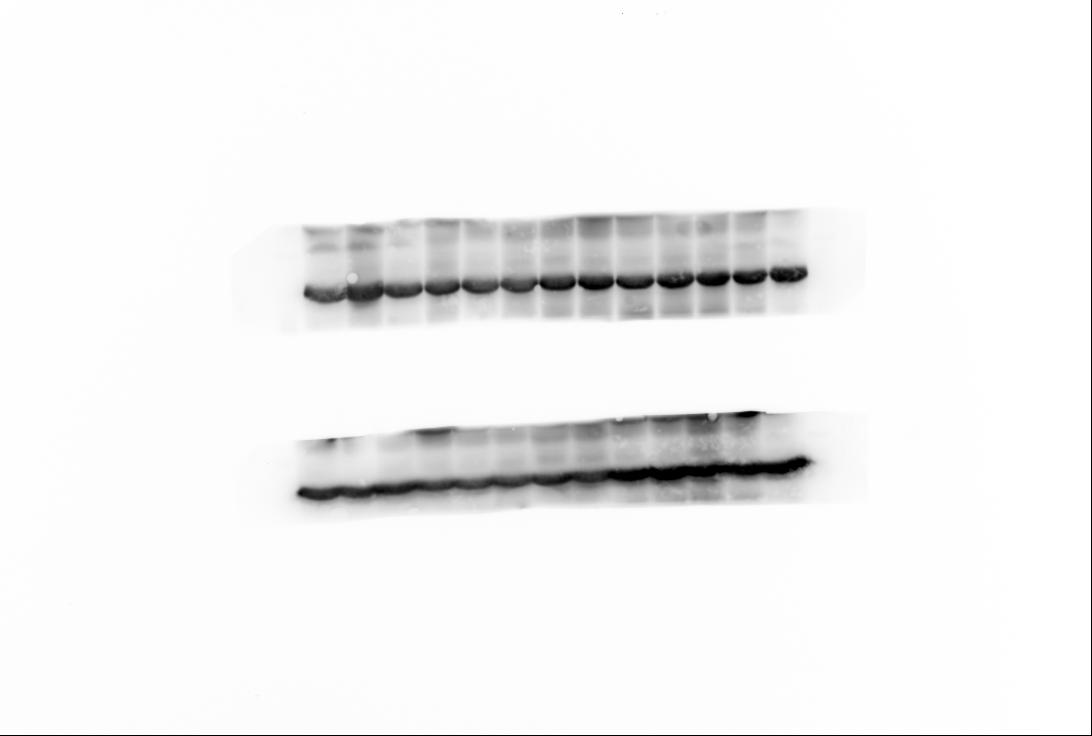

Supplement: Supplementary file 1 [file DataSheet_1.zip › original blots figures/original blots figures/5C/GAPDH-34.TIF]

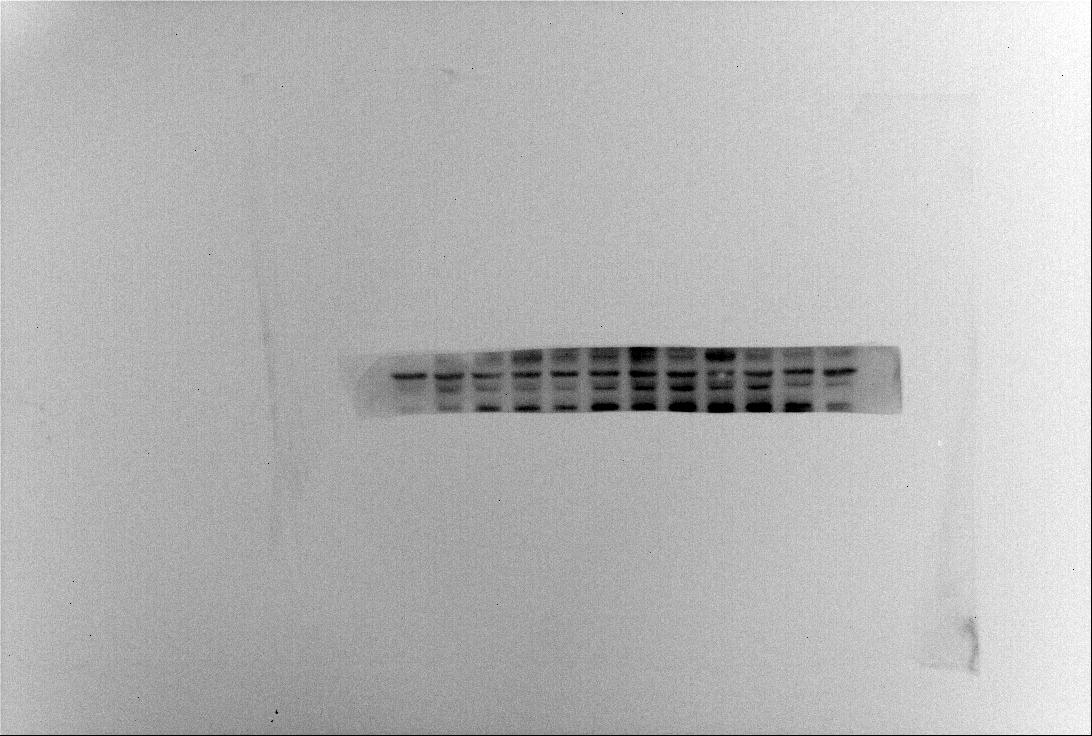

Supplement: Supplementary file 1 [file DataSheet_1.zip › original blots figures/original blots figures/5C/GAPDH-6.TIF]

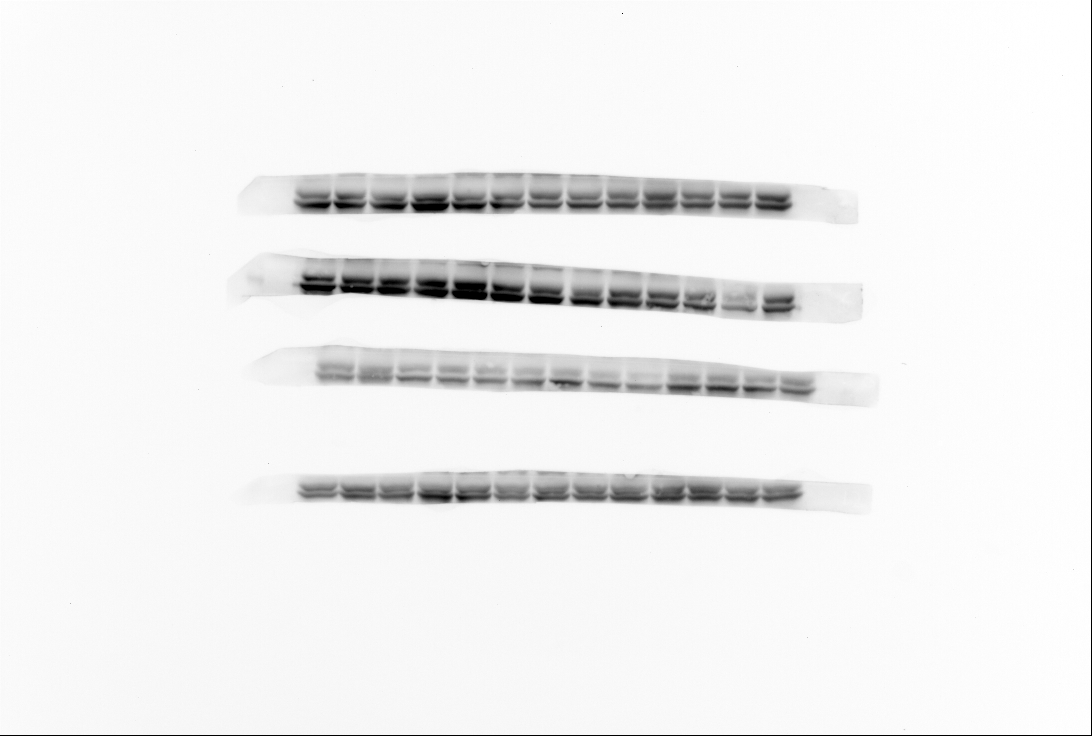

Supplement: Supplementary file 1 [file DataSheet_1.zip › original blots figures/original blots figures/5C/IKK1-4.TIF]

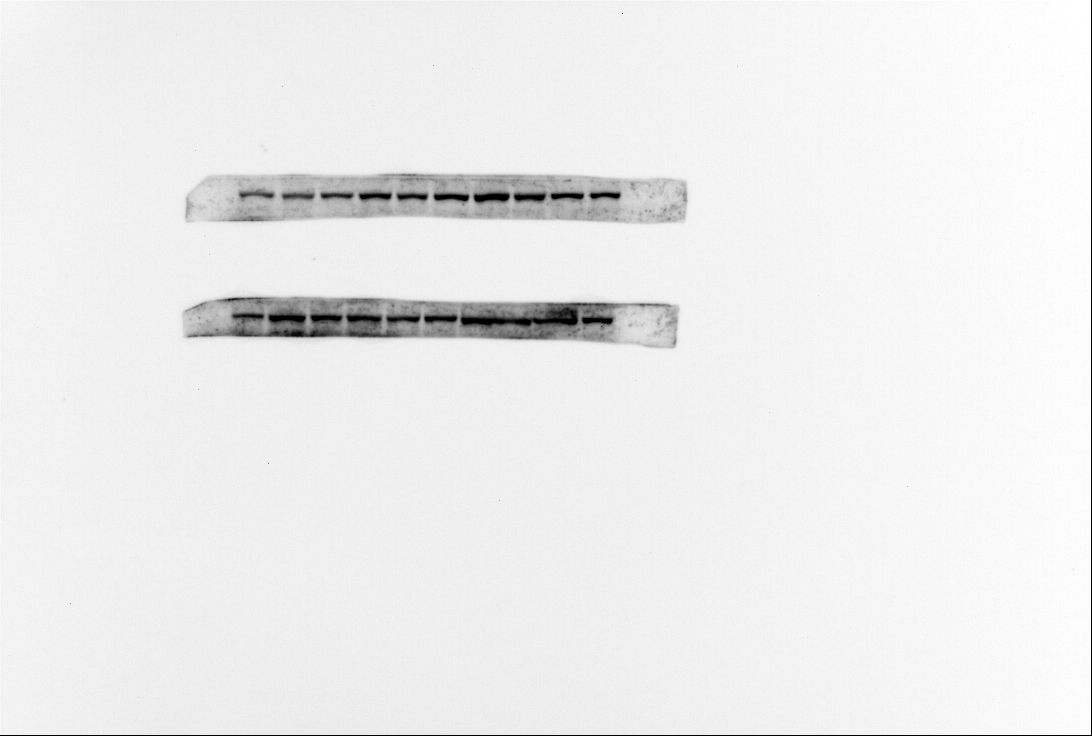

Supplement: Supplementary file 1 [file DataSheet_1.zip › original blots figures/original blots figures/5C/NFKB-12.TIF]

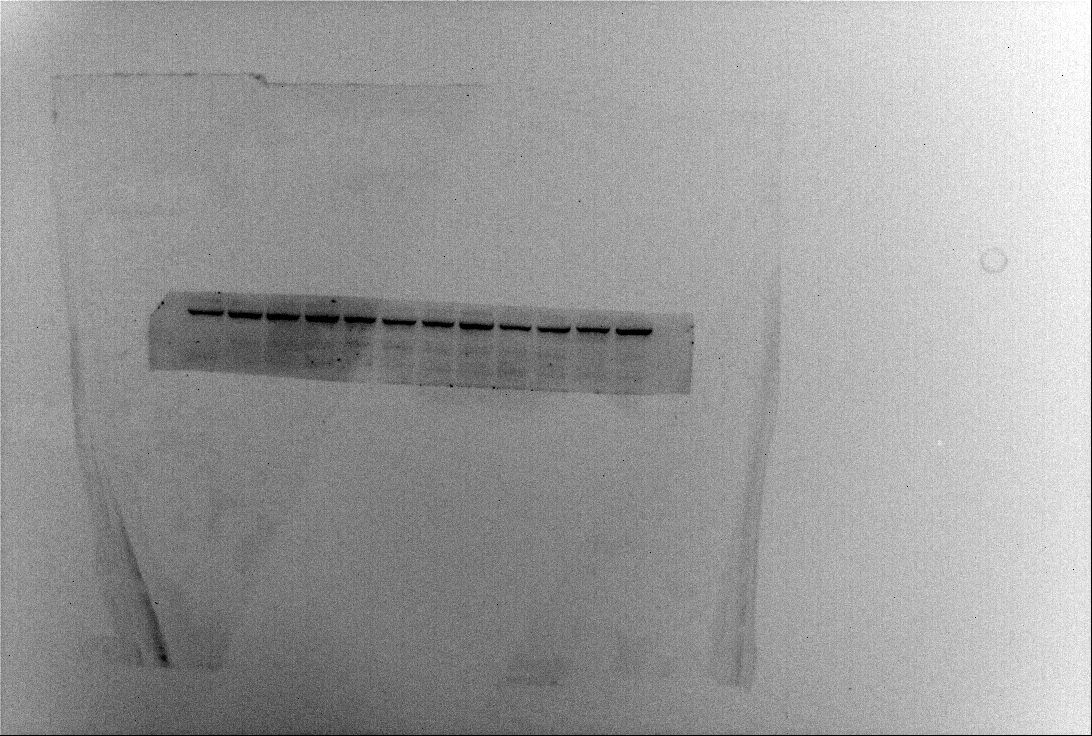

Supplement: Supplementary file 1 [file DataSheet_1.zip › original blots figures/original blots figures/5C/NFKB-6.TIF]

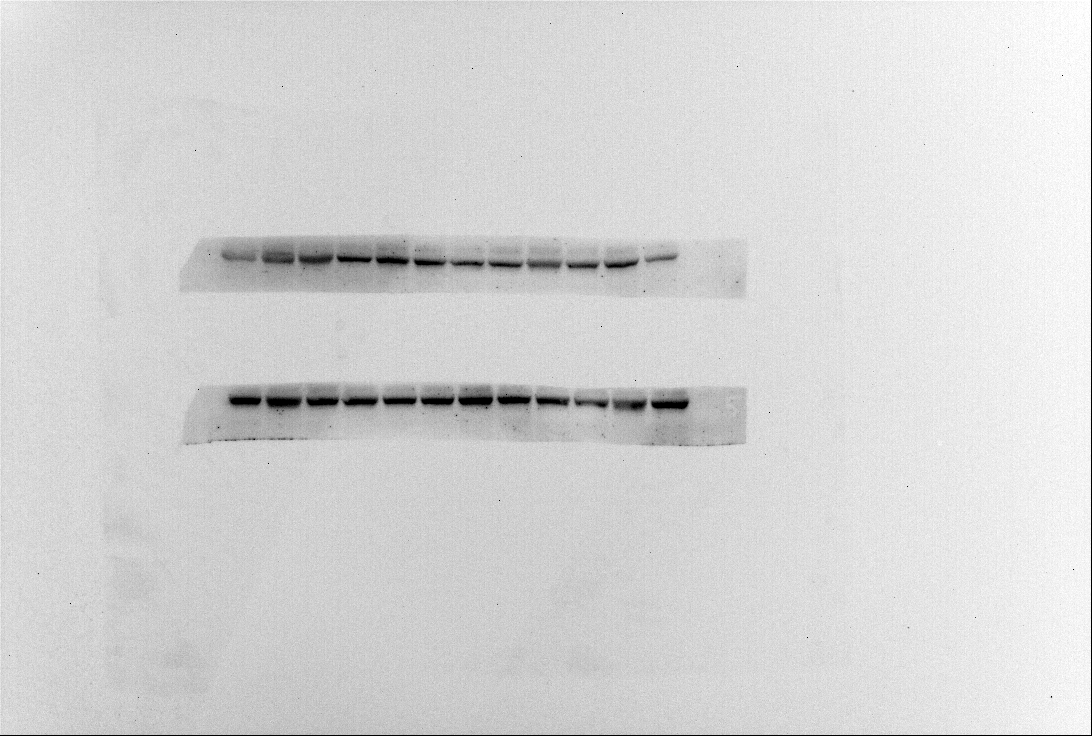

Supplement: Supplementary file 1 [file DataSheet_1.zip › original blots figures/original blots figures/5C/NF-KB-ZH.tif]

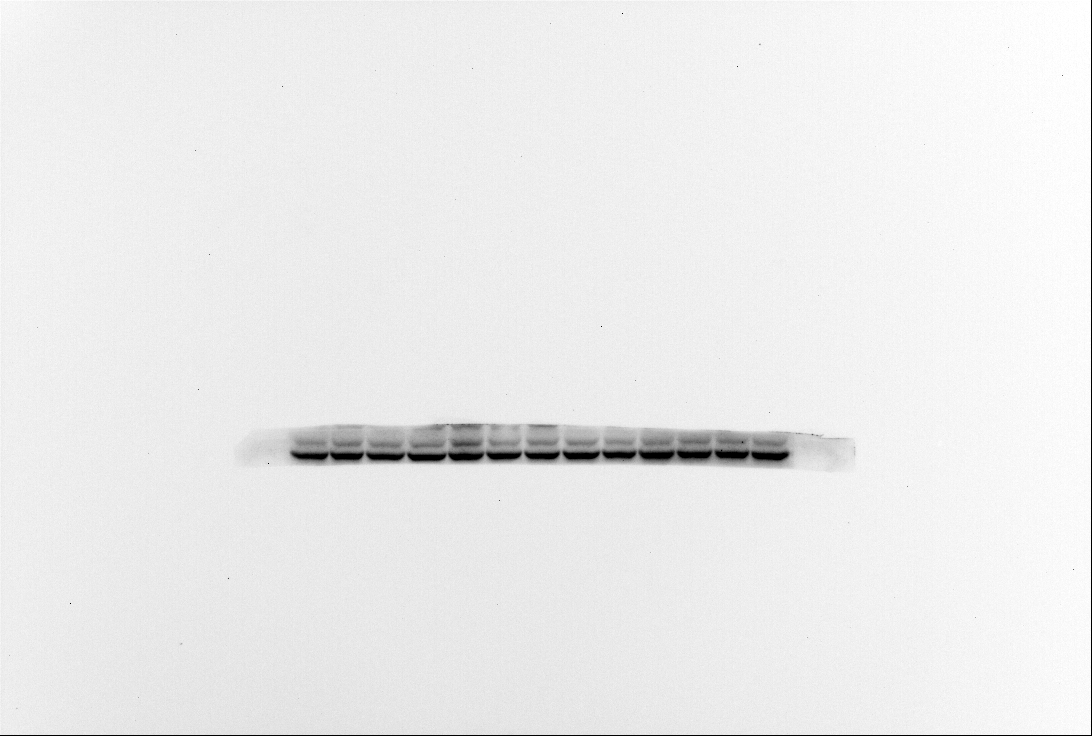

Supplement: Supplementary file 1 [file DataSheet_1.zip › original blots figures/original blots figures/5C/p-IKK-1.TIF]

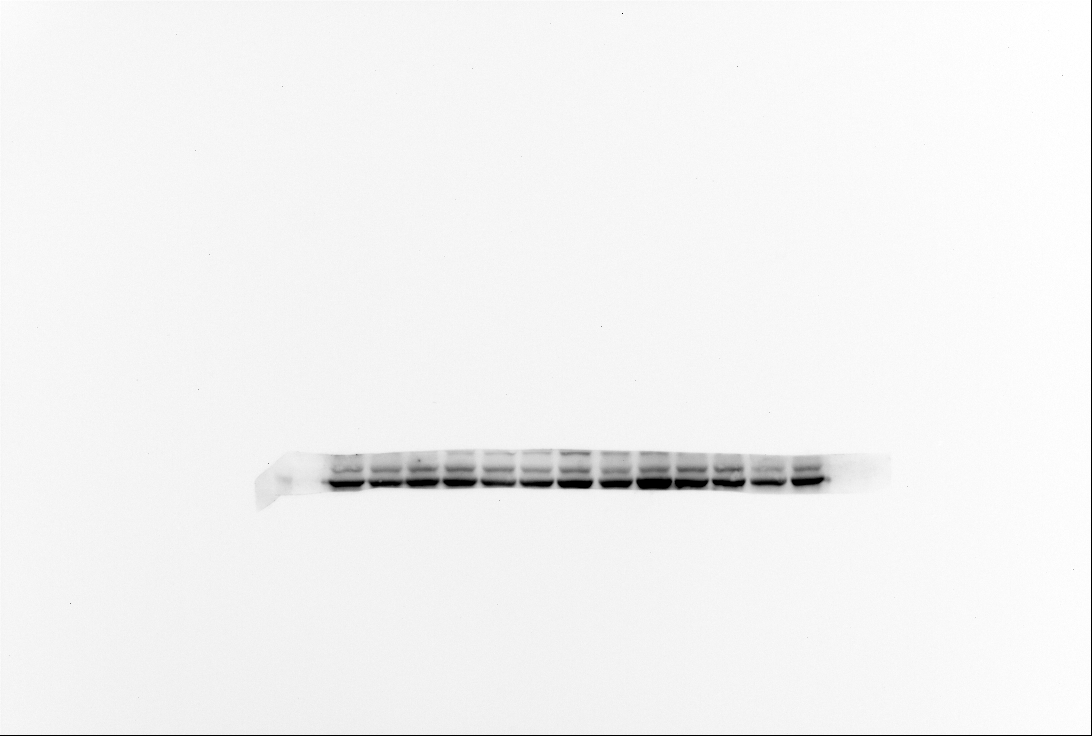

Supplement: Supplementary file 1 [file DataSheet_1.zip › original blots figures/original blots figures/5C/p-IKK-2.TIF]

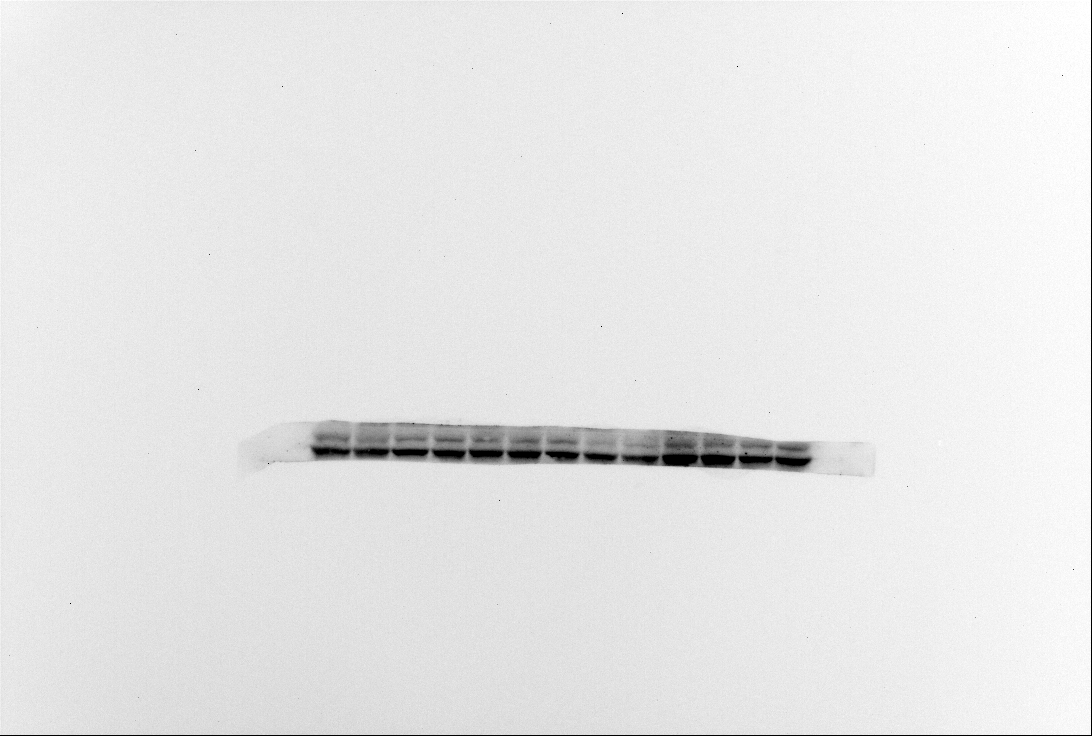

Supplement: Supplementary file 1 [file DataSheet_1.zip › original blots figures/original blots figures/5C/p-IKK-3.TIF]

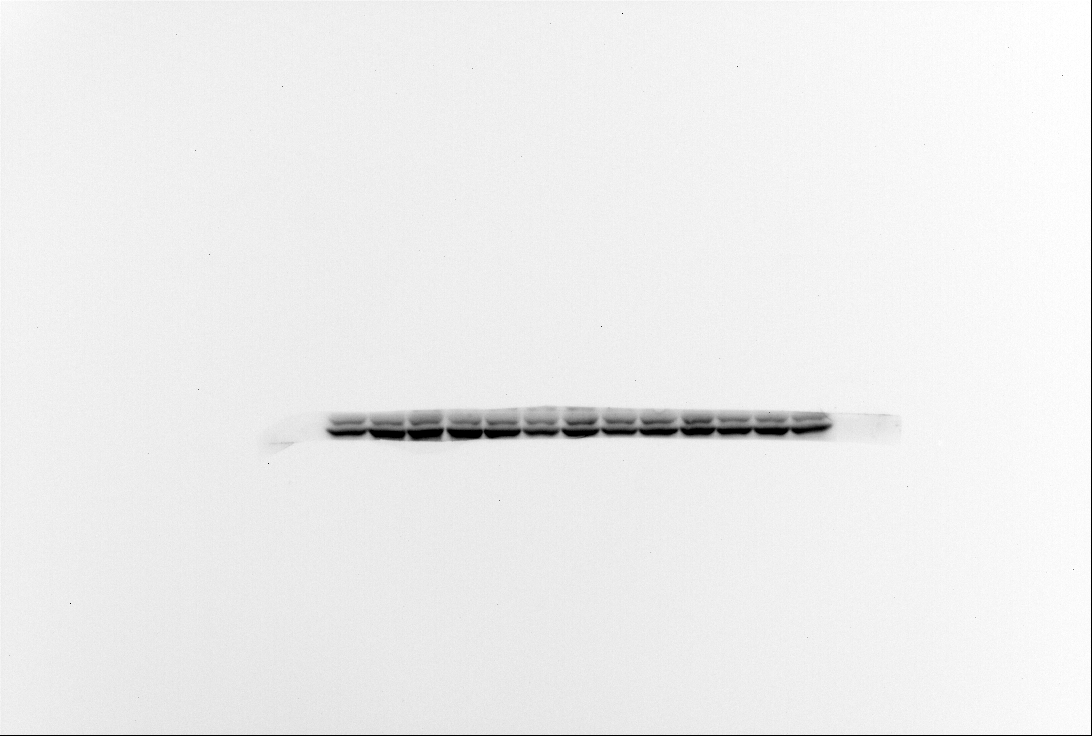

Supplement: Supplementary file 1 [file DataSheet_1.zip › original blots figures/original blots figures/5C/p-IKK-4.TIF]

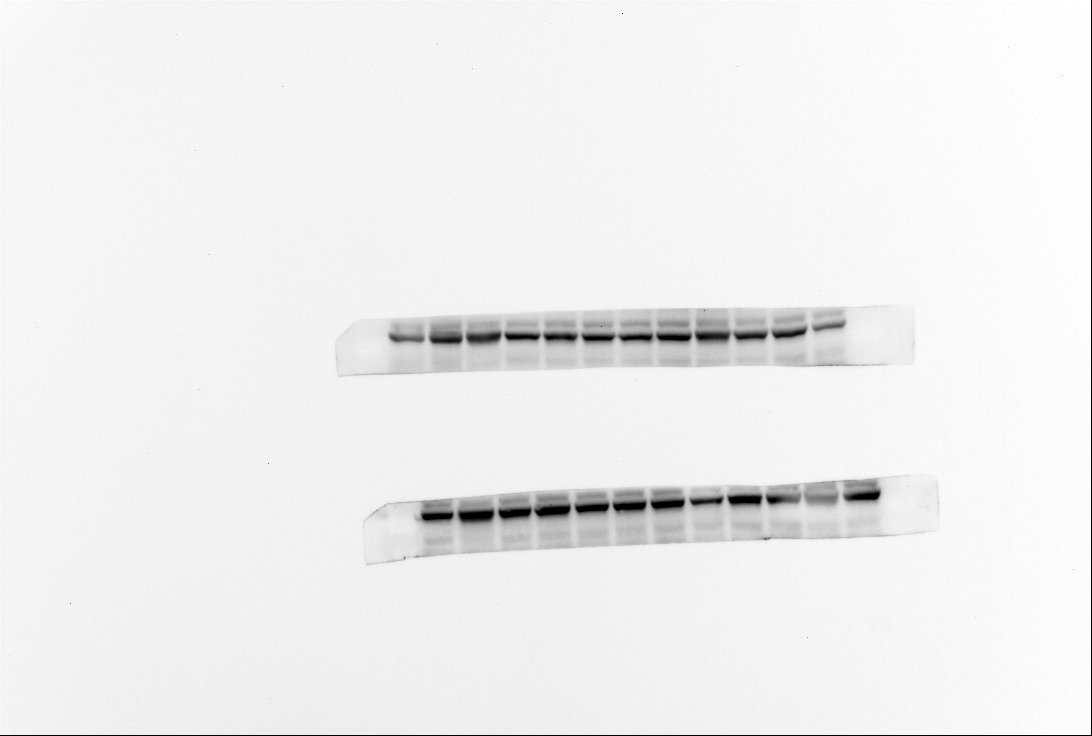

Supplement: Supplementary file 1 [file DataSheet_1.zip › original blots figures/original blots figures/5C/p-NFkB.TIF]

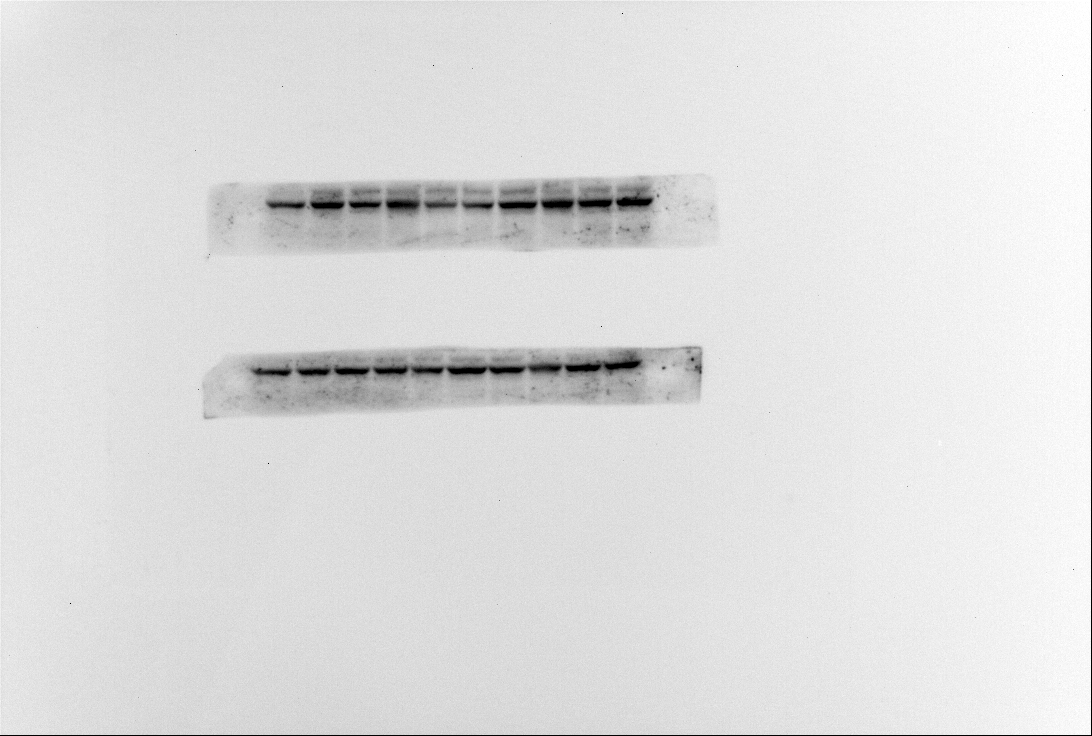

Supplement: Supplementary file 1 [file DataSheet_1.zip › original blots figures/original blots figures/5C/p-NF-KB-12.TIF]

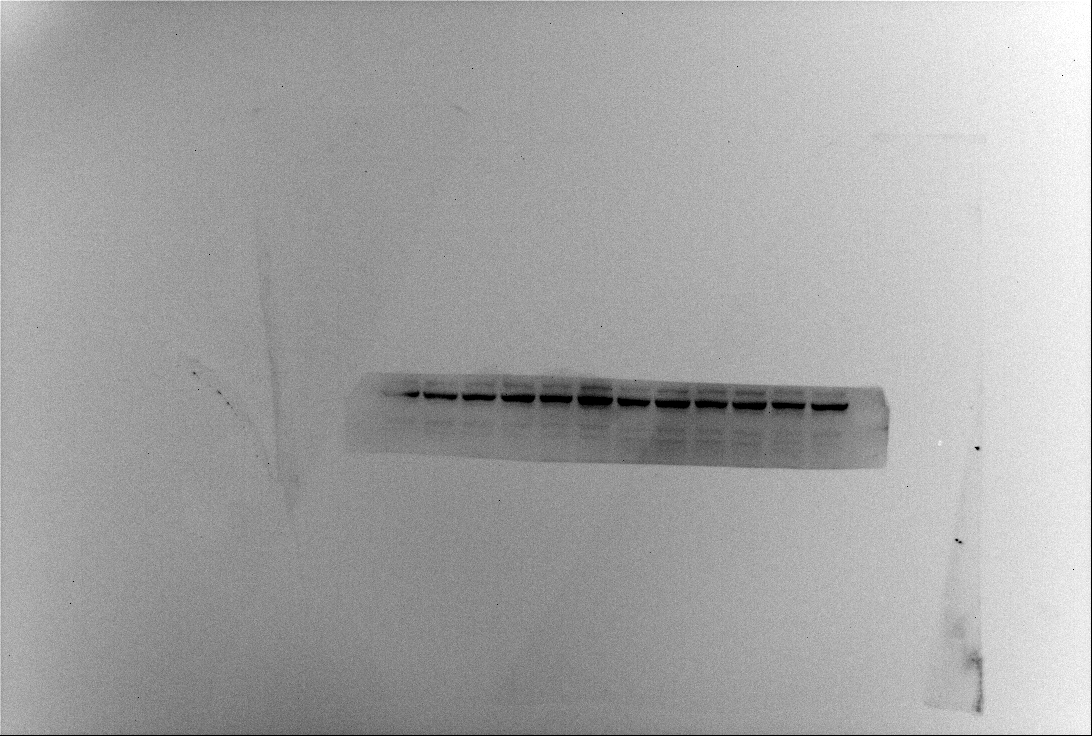

Supplement: Supplementary file 1 [file DataSheet_1.zip › original blots figures/original blots figures/5C/p-NFKB-6.TIF]

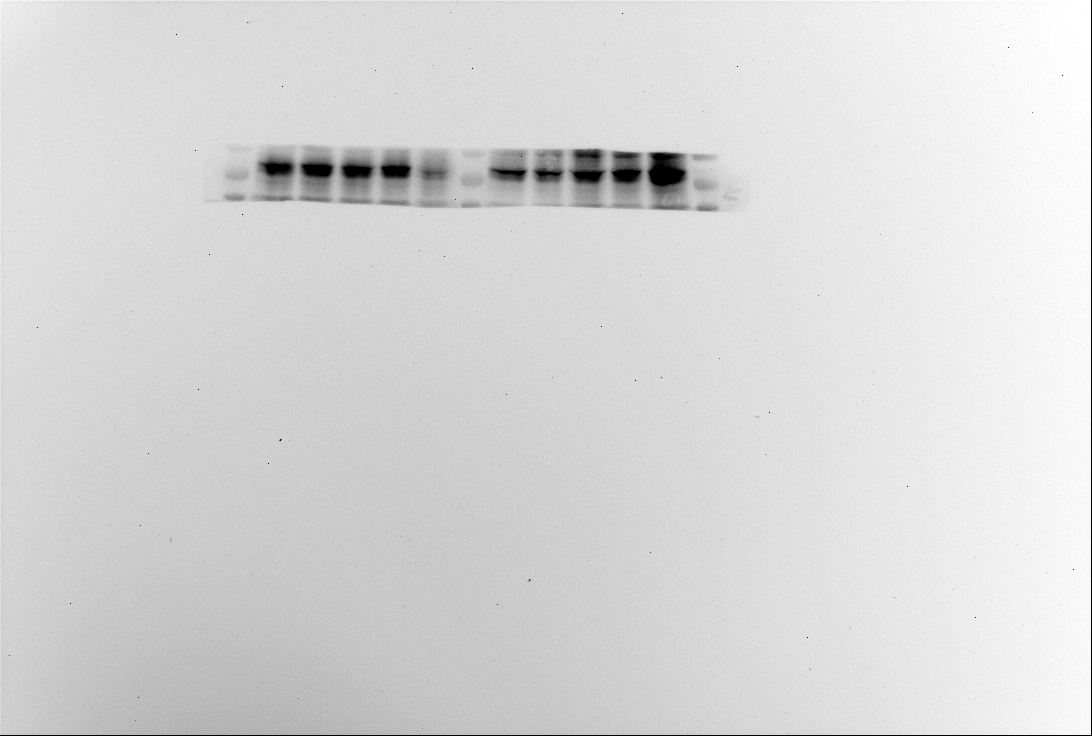

Supplement: Supplementary file 1 [file DataSheet_1.zip › original blots figures/original blots figures/7/ERM-1.TIF]

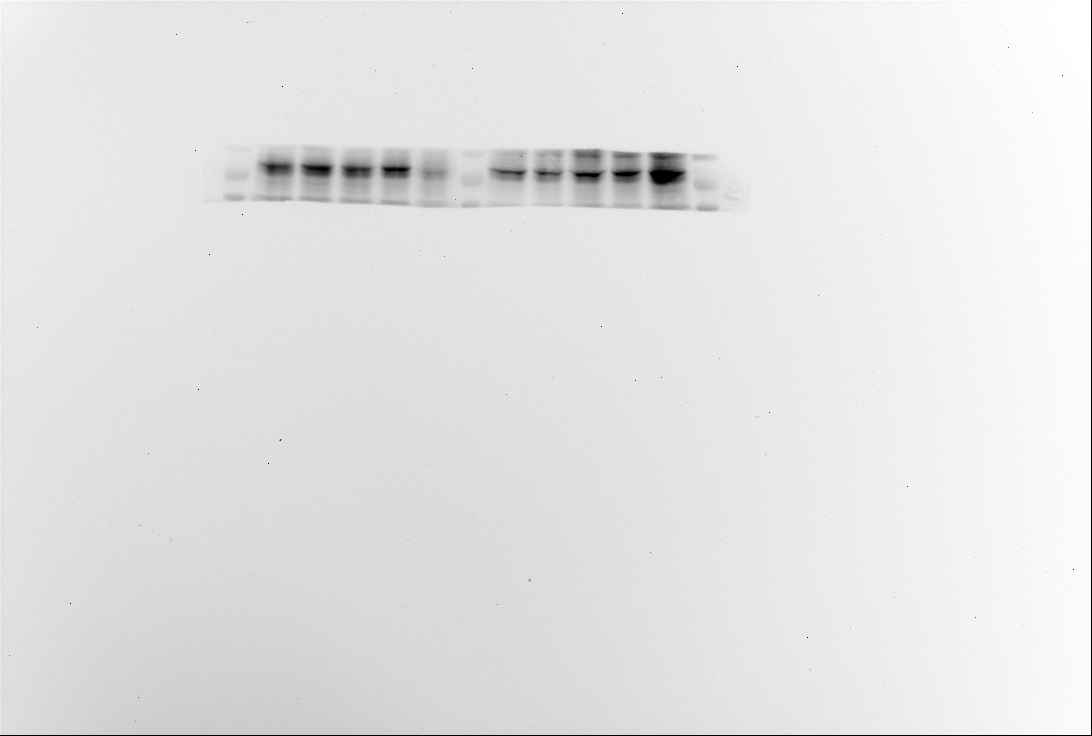

Supplement: Supplementary file 1 [file DataSheet_1.zip › original blots figures/original blots figures/7/ERM-2.TIF]

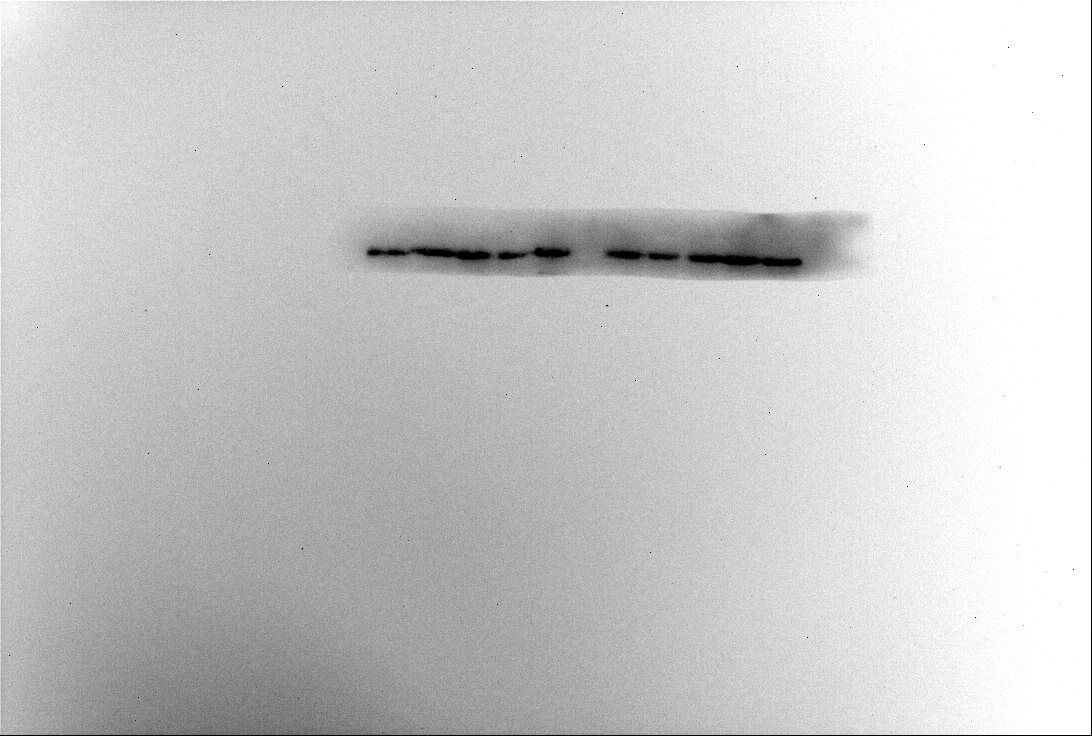

Supplement: Supplementary file 1 [file DataSheet_1.zip › original blots figures/original blots figures/7/MLC-1.TIF]

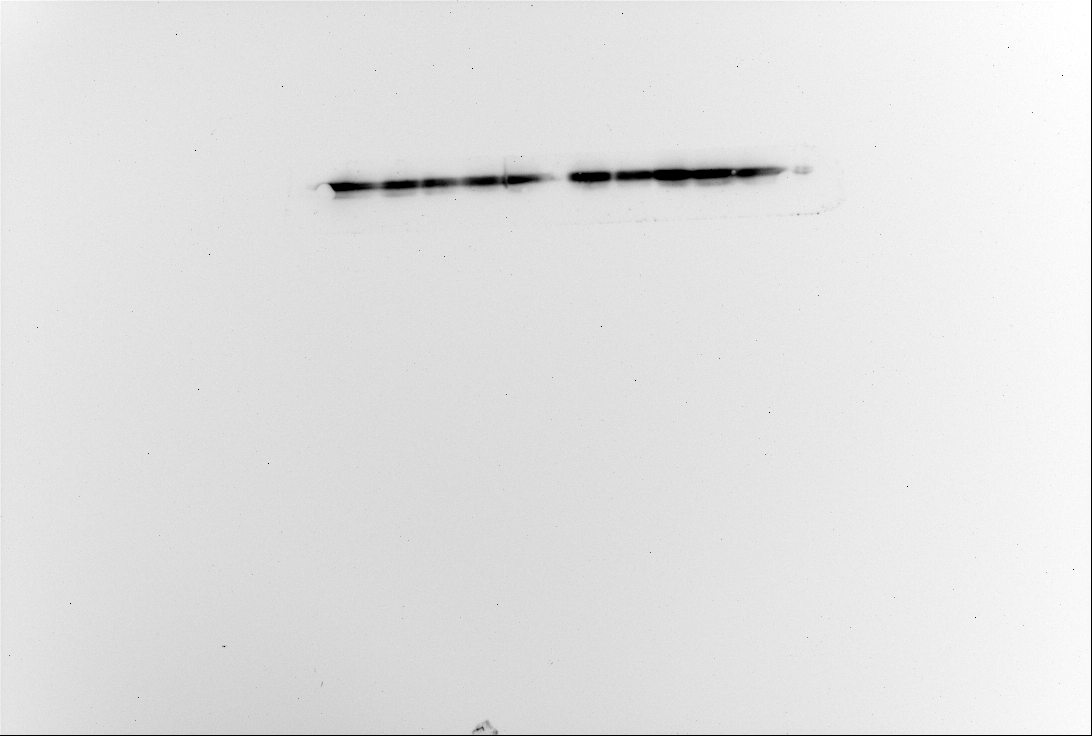

Supplement: Supplementary file 1 [file DataSheet_1.zip › original blots figures/original blots figures/7/MLC-2.tif]

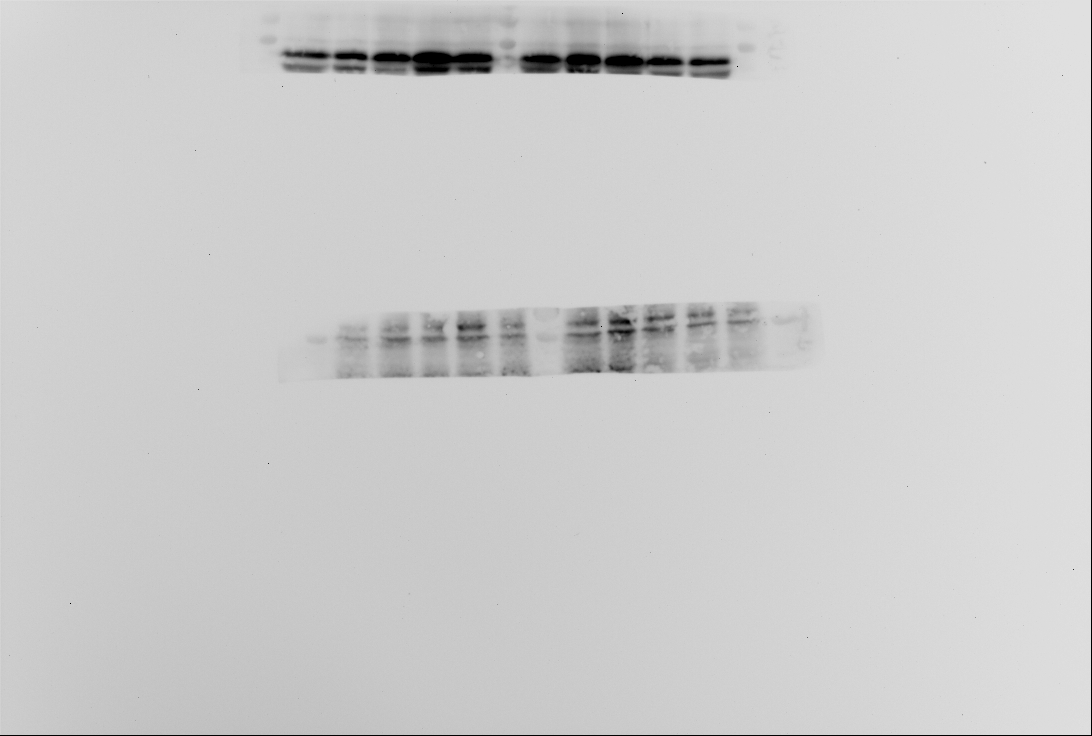

Supplement: Supplementary file 1 [file DataSheet_1.zip › original blots figures/original blots figures/7/MYPT1-1.TIF]

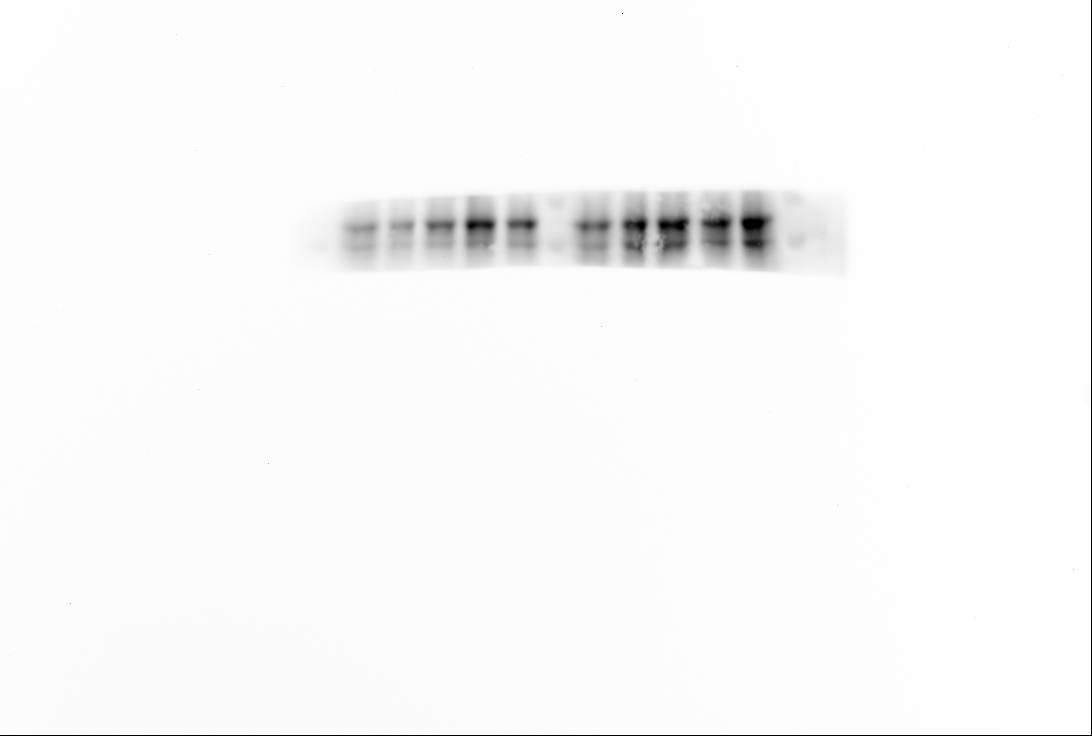

Supplement: Supplementary file 1 [file DataSheet_1.zip › original blots figures/original blots figures/7/MYPT1-2.TIF]

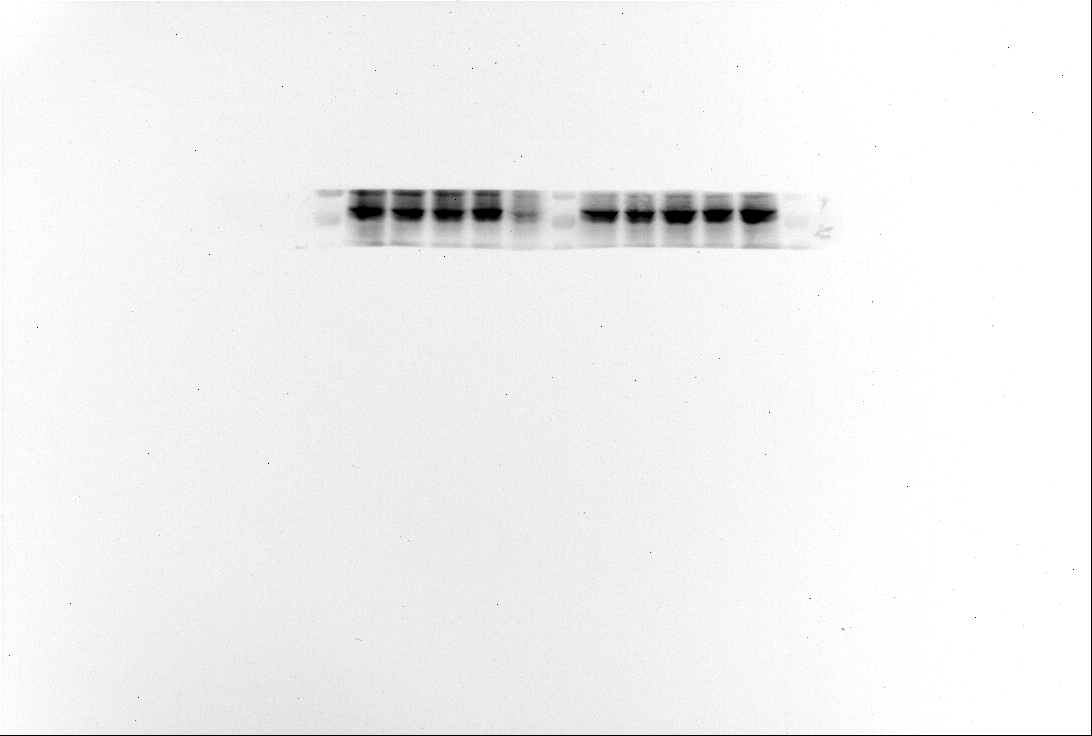

Supplement: Supplementary file 1 [file DataSheet_1.zip › original blots figures/original blots figures/7/p-ERM-1.TIF]

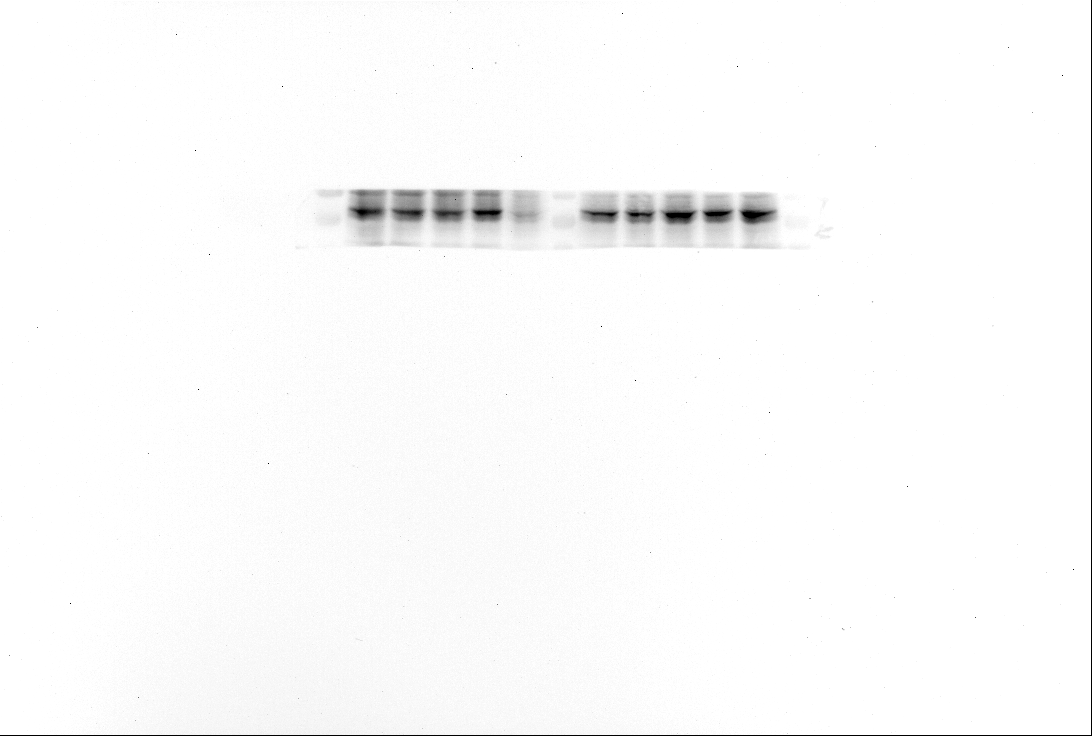

Supplement: Supplementary file 1 [file DataSheet_1.zip › original blots figures/original blots figures/7/p-ERM-2.TIF]

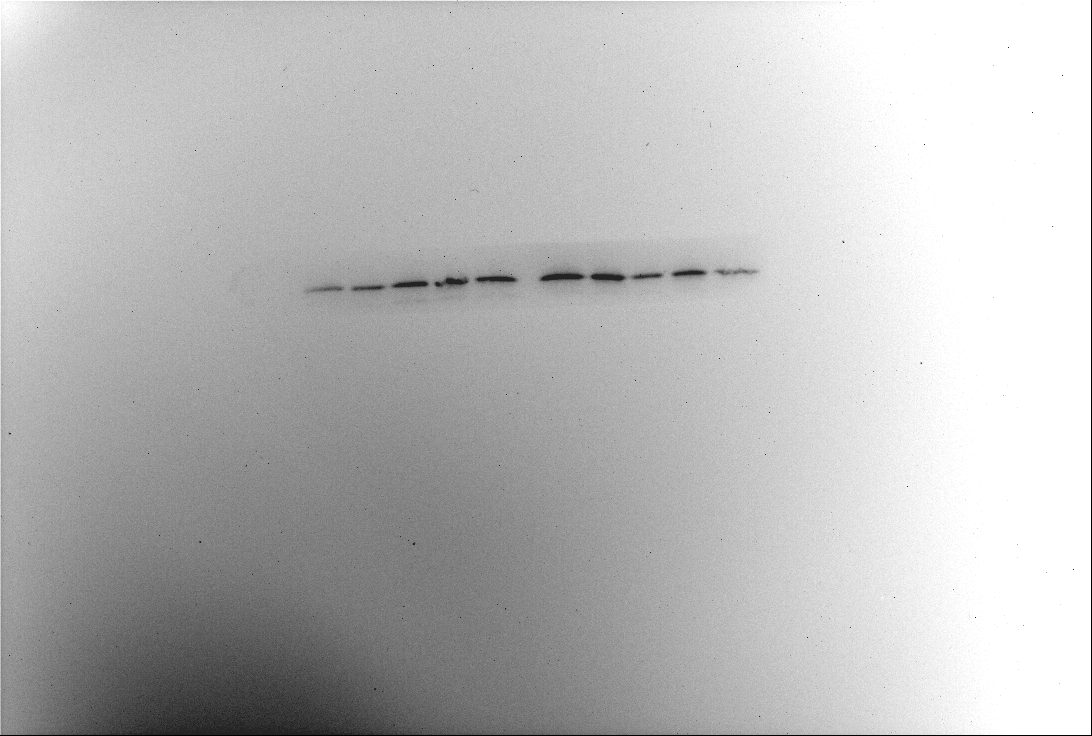

Supplement: Supplementary file 1 [file DataSheet_1.zip › original blots figures/original blots figures/7/p-MLC-1.TIF]

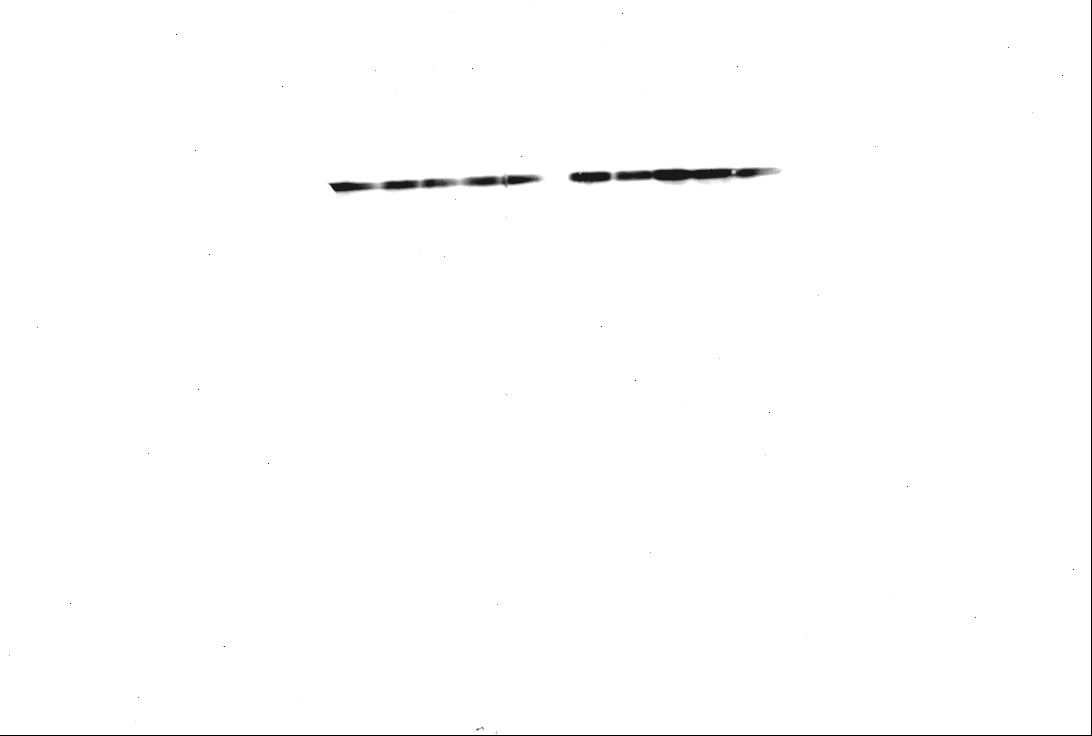

Supplement: Supplementary file 1 [file DataSheet_1.zip › original blots figures/original blots figures/7/p-MLC-2.tif]

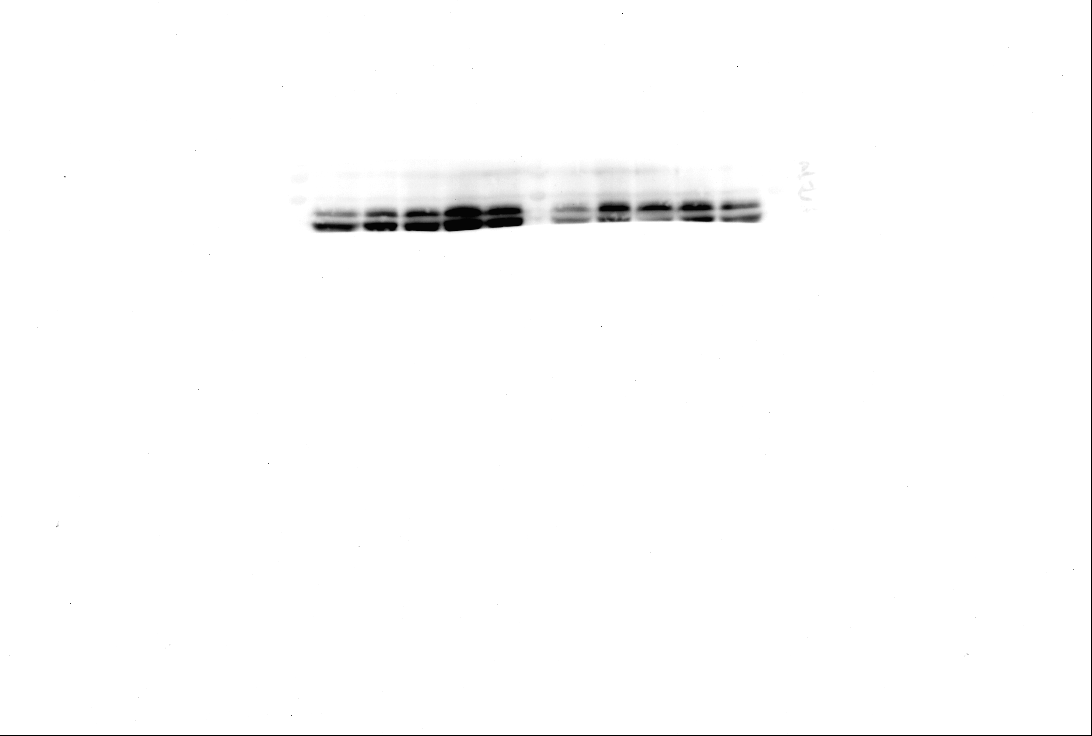

Supplement: Supplementary file 1 [file DataSheet_1.zip › original blots figures/original blots figures/7/p-MYPT1-1.TIF]

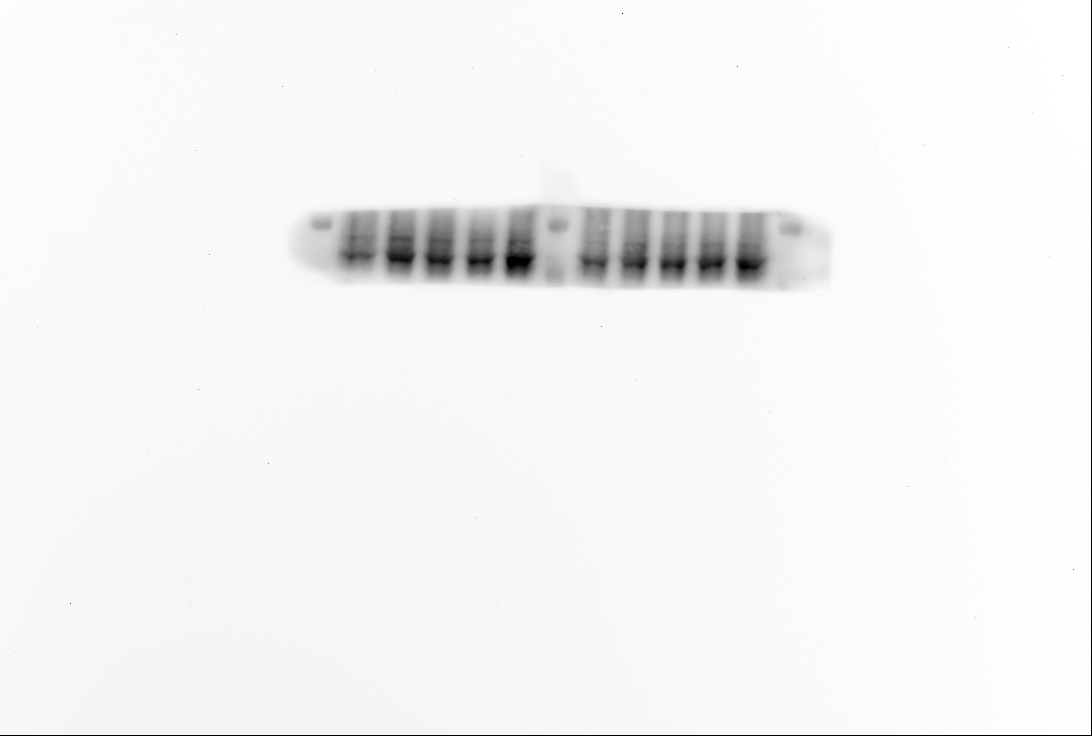

Supplement: Supplementary file 1 [file DataSheet_1.zip › original blots figures/original blots figures/7/p-MYPT1-2.TIF]

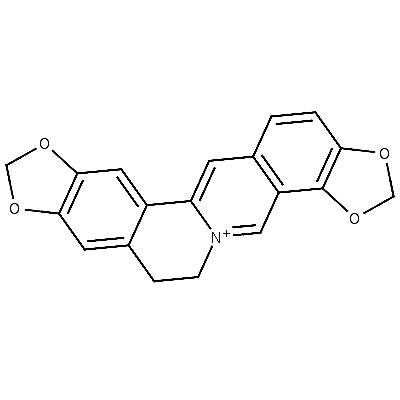

Supplement: Supplementary file 3 [file Image_1.png]

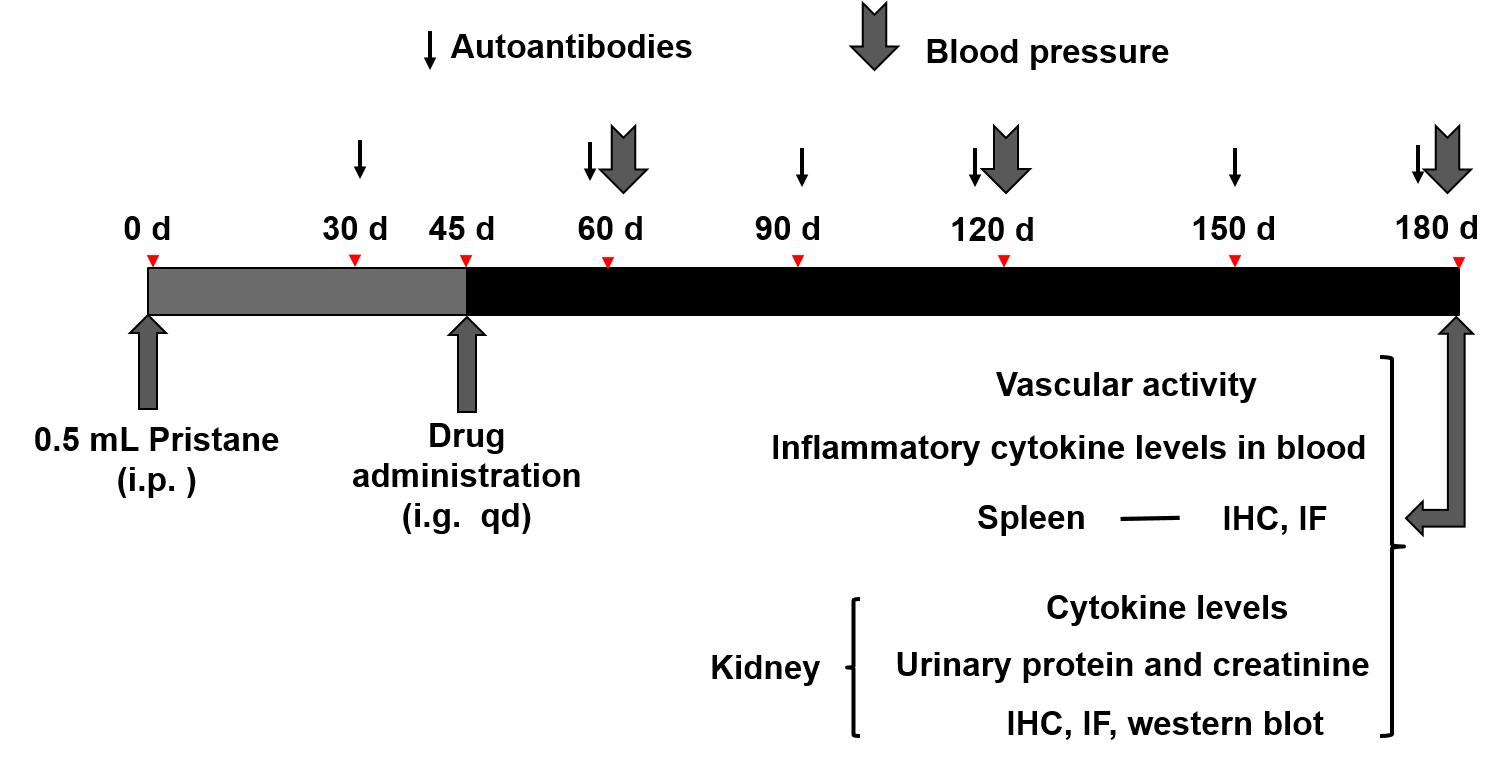

Supplement: Supplementary file 4 [file Image_2.tif]

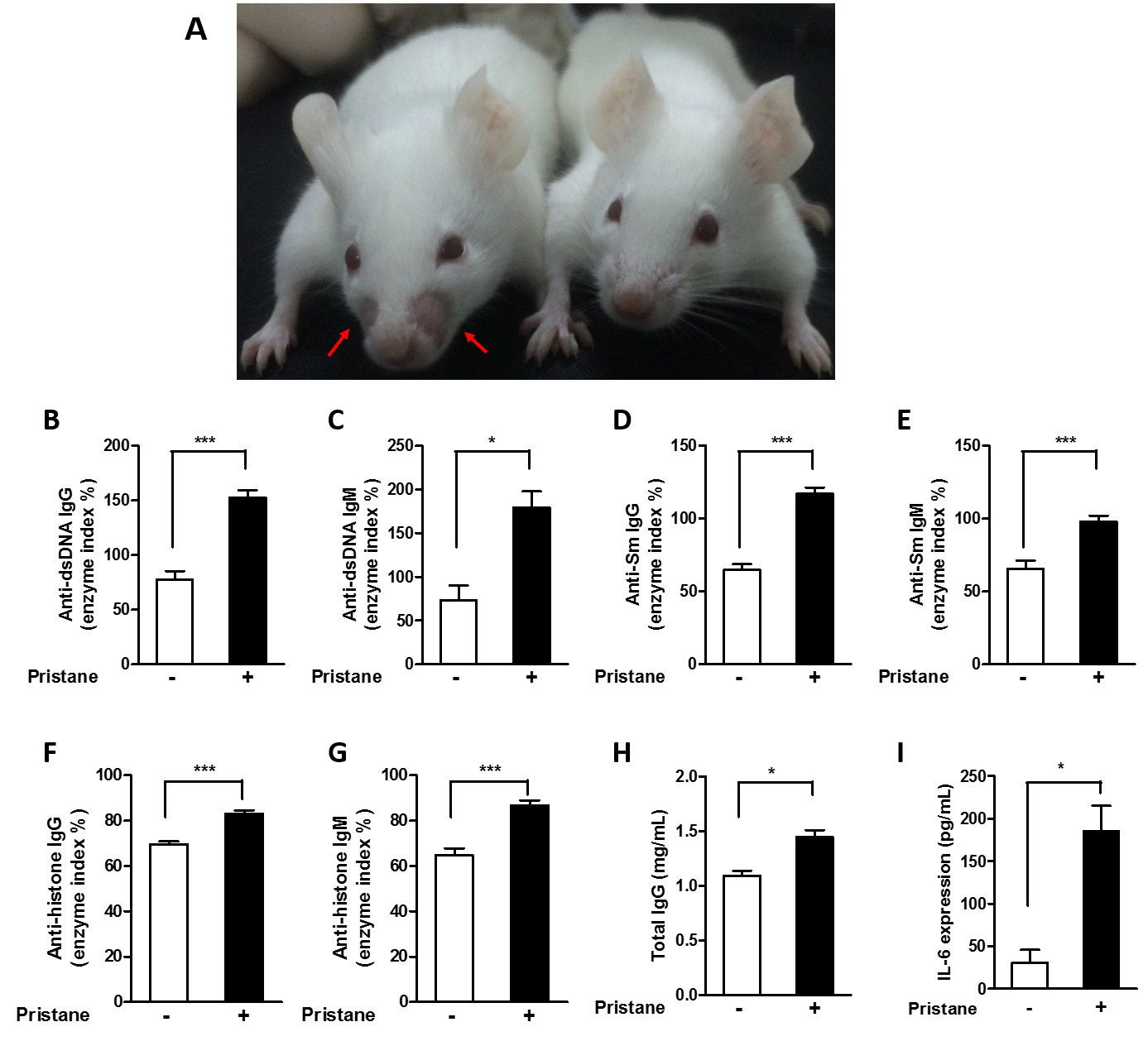

Supplement: Supplementary file 5 [file Image_3.tif]

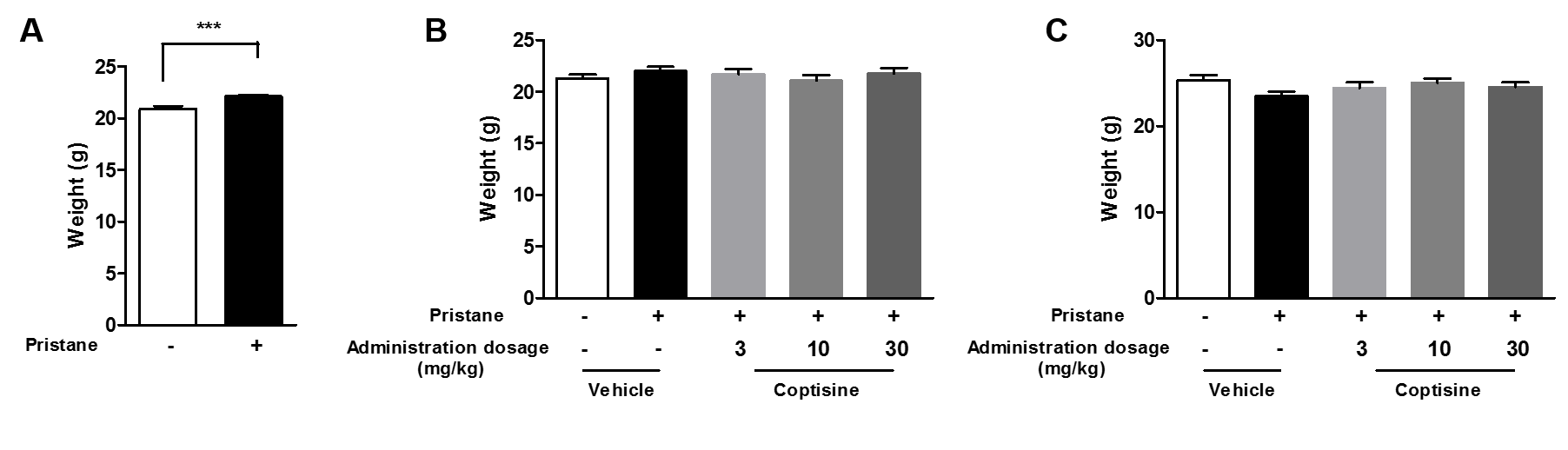

Supplement: Supplementary file 6 [file Image_4.tif]

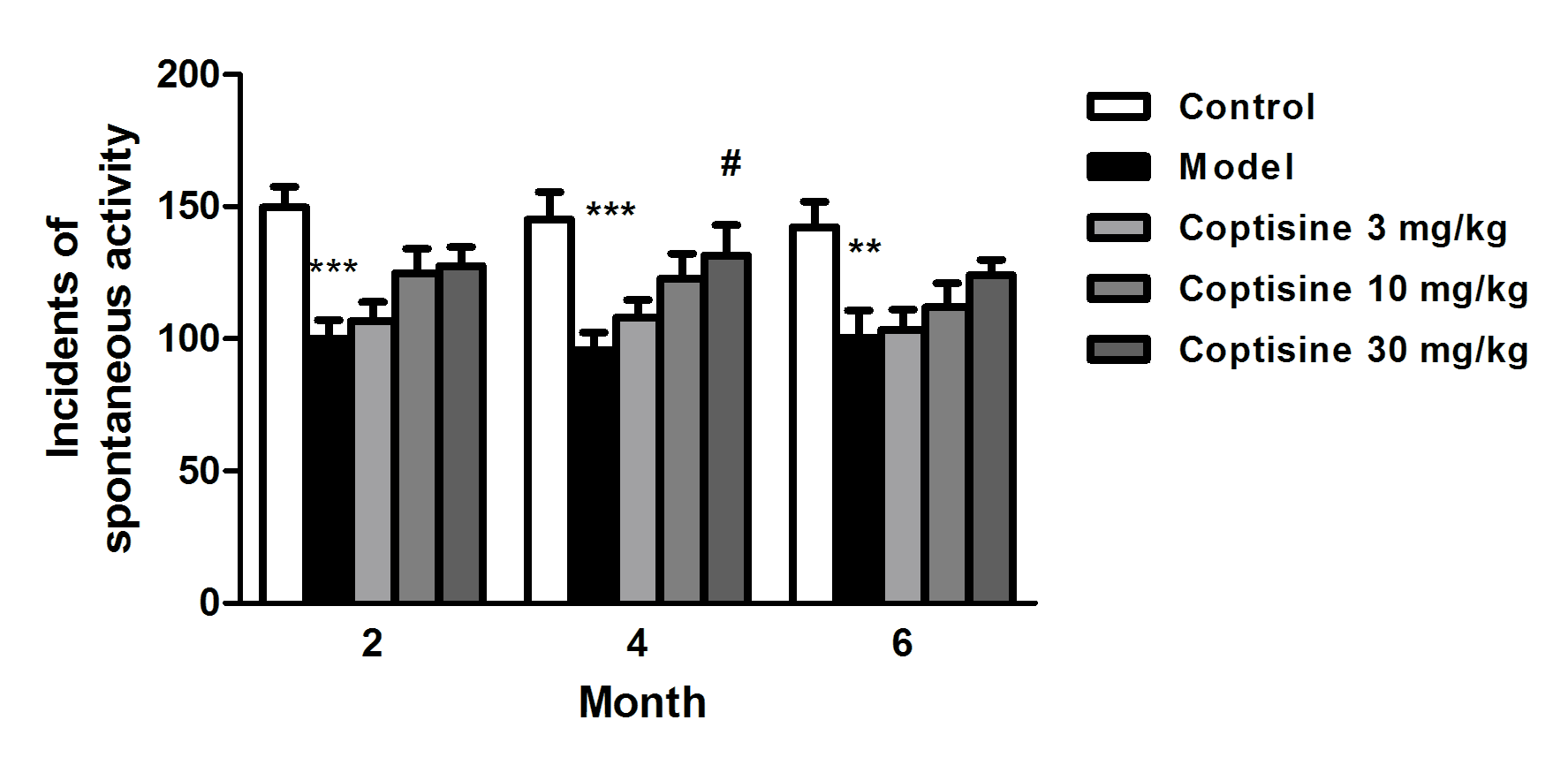

Supplement: Supplementary file 7 [file Image_5.tif]
